# PAWI-2 overcomes tumor stemness and drug resistance via cell cycle arrest in integrin β_3_-KRAS-dependent pancreatic cancer stem cells

**DOI:** 10.1038/s41598-020-65804-5

**Published:** 2020-06-08

**Authors:** Jiongjia Cheng, John R. Cashman

**Affiliations:** Human BioMolecular Research Institute and ChemRegen, Inc., San Diego, CA 92121 USA

**Keywords:** Cancer stem cells, Cancer therapy, Drug discovery

## Abstract

Today, pancreatic cancer (PC) remains a major health problem in the US. The fact that cancer stem cells (CSCs) become enriched in humans following anti-cancer therapy implicates CSCs as key contributors to tumor dormancy, metastasis, and relapse in PC. A highly validated CSC model (FGβ_3_ cells) was used to test a novel compound (PAWI-2) to eradicate CSCs. Compared to parental bulk FG cells, PAWI-2 showed greater potency to inhibit cell viability and self-renewal capacity of FGβ_3_ cells. For FGβ_3_ cells, dysregulated integrin β_3_-KRAS signaling drives tumor progression. PAWI-2 inhibited β_3_-KRAS signaling independent of KRAS. This is clinically relevant. PAWI-2 targeted the downstream TBK1 phosphorylation cascade that was negatively regulated by optineurin phosphorylation via a feedback mechanism. This was confirmed by TBK1 genetic knockdown or co-treatment with TBK1-specific inhibitor (MRT67307). PAWI-2 also overcame erlotinib (an EGFR inhibitor) resistance in FGβ_3_ cells more potently than bortezomib. In the proposed working model, optineurin acts as a key regulator to link inhibition of KRAS signaling and cell cycle arrest (G2/M). The findings show PAWI-2 is a new approach to reverse tumor stemness that resensitizes CSC tumors to drug inhibition.

## Introduction

Pancreatic cancer (PC) remains a major health problem in the US and soon will be the second most common cause of mortality due to cancer^1,2^. One of the only curable treatment options for PC is surgical resection^3^. However, disease recurrence is still at high risk after surgery and a majority of post-surgical patients develop advanced metastatic disease, thus necessitating chemo- and radiation therapies^4^. Front-line chemotherapies cause serious side effects^5–7^. A majority of PC patients are often resistant to clinical therapies^4^. Thus, it remains a challenge to develop an efficacious clinically useful PC therapy.

Cancer stem cells (CSCs) are hallmarks of cancer and inherently resistant to medical therapy^8,9^. CSCs become enriched in humans following chemo- or radiotherapy. This implicates CSCs as key contributors to tumor dormancy, metastasis, and relapse^10,11^. These functional features of CSCs make CSCs different from bulk tumor cells and enable CSCs to initiate and maintain tumor development from tumor cells present in a malignant tumor^12,13^.

CSCs were identified and prospectively isolated from a number of solid tumors by using CSC-specific biomarkers^12,13^. These biomarkers show a distinct cell population with increased renewal capacity and the ability to recapitulate heterogeneity, multi-lineage differentiation and long-term repopulation^12,13^. One cell surface adhesion molecule (i.e., integrin α_v_β_3_) is well-established as a driver of tumor progression due to association with greater incidence of metastasis^14,15^. This occurs in a variety of cancers^15–17^. The capability of integrin α_v_β_3_ to trigger anchorage-independent cell survival and tumor metastasis^14,18,19^ shows that integrin α_v_β_3_ expression is a biomarker/functional contributor to CSC progression and drug resistance. Human pancreatic cancer stem cells (hPCSCs) reported previously (i.e., FGβ_3_ cells) are a validated human CSC model^19–21^ that overexpresses integrin α_v_β_3._ In FGβ_3_ cells, integrin α_v_β_3_ recruits Kirsten rat sarcoma viral oncogene homologue GTPase (KRAS) and RAS Like Proto-Oncogene B (RalB) to activate serine/threonine kinase Tank-binding kinase 1(TBK1, IκB kinase (IKK)-related kinase) and nuclear factor kappa-light-chain-enhancer of activated B cells (NF-κB) to trigger dysregulated KRAS-RalB-NF-κB. This pathway was reported to be a pharmacological target to reverse CSC-like properties or re-sensitize drug resistance for established FGβ_3_ tumors^19–21^.

**Figure 1:**
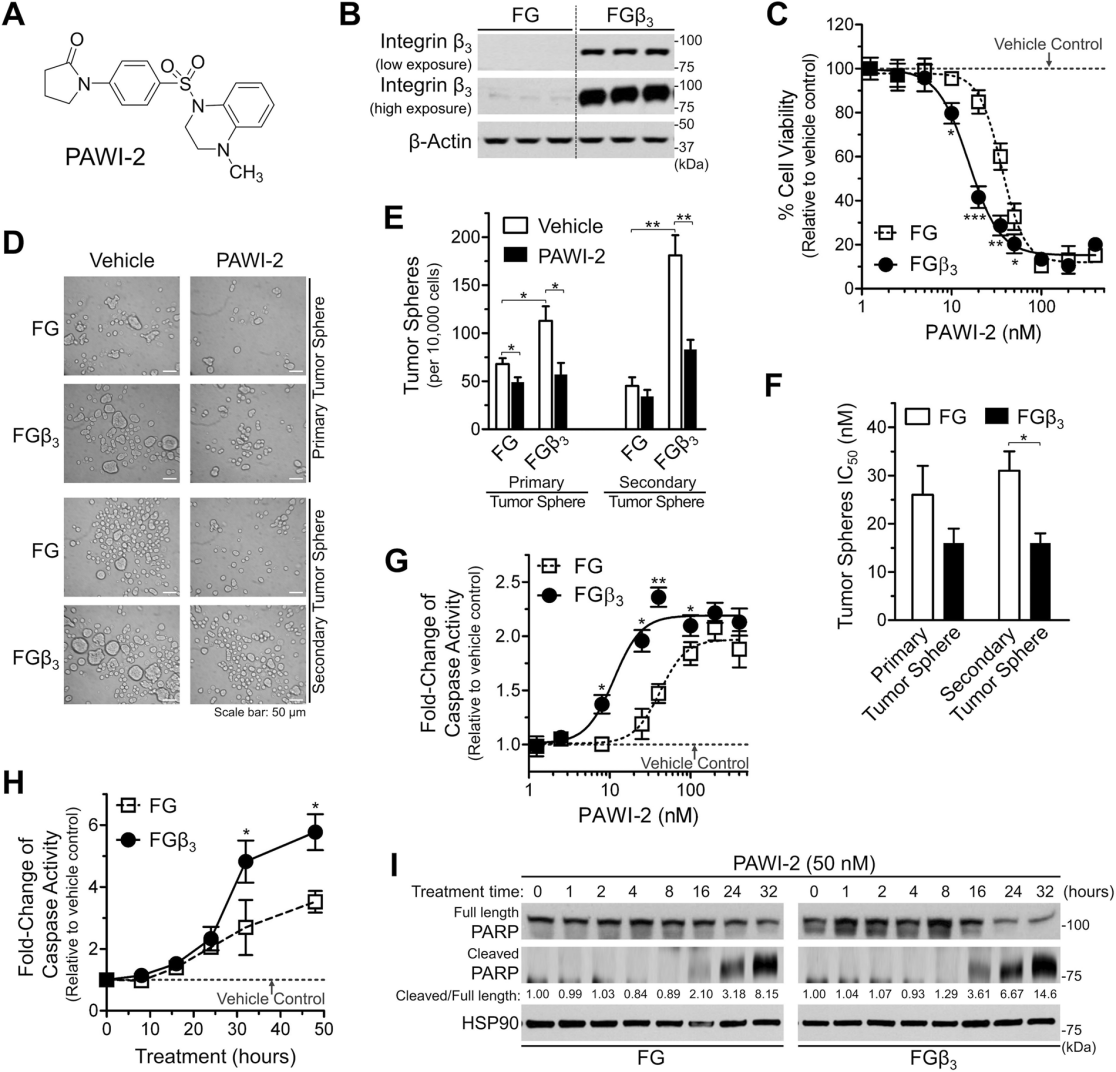
PAWI-2 overcomes tumor stemness driven by integrin β_3_ expression. **(A**) Chemical structure of PAWI-2. (**B**) Overexpression of integrin β_3_ in FGβ_3_ cells (with human β_3_/pcDNA3.1 vector) compared to parental FG cells (with empty vector). (**C**) Dose-dependent effect of PAWI-2 on inhibition of cell viability in FG and FGβ_3_ cells. (**D–F**) Effect of PAWI-2 on the inhibition of primary and secondary tumor sphere formation in FG and FGβ_3_ cells: (**D**) representative tumor sphere images; (**E**) self-renewal capacity measured by quantifying the number of primary and secondary tumor spheres; (**F**) bar graph of the half-maximum inhibitory concentrations (IC_50_s). (**G–I**) Effect of PAWI-2 on activation of cell apoptosis in FG and FGβ_3_ cells: (**G**) dose-dependent and (**H**) time-dependent activation of caspase-3/7 activity by PAWI-2 determined by Caspase-Glo 3/7 assay; **(I**) immunoblot analyses of PARP (full length) and cleaved PARP as determined with whole-cell extracts. Concentration of PAWI-2 used were as indicated: 1.2–400 nM in **C**, **G**, 20 nM in **D**, **E** and 50 nM in **H**, **I**; treatment time used was as indicated: 72 hours in **C**, 24 hours in **D-G**, 0–48 hours in **H** and 0–32 hours in **I**; vehicle control (0.5% DMSO). β-Actin or HSP90 were used as loading controls in **B**, **I**. Data were mean ± SD (n = 3) in **C**, **E–H**; *P*-values were estimated by Student *t* tests in **C**, **E–H** (*P < 0.05, **P < 0.01, ***P < 0.001). The full-length blots are presented in Supplementary Fig. S7.

Given the important role of hPCSCs, a novel treatment strategy that targets hPCSCs or their extrinsic and intrinsic regulators could be of significant clinical utility to treat PC. Herein, we report PAWI-2 (Fig. 1A) that kills drug-resistant hPCSCs (i.e., FGβ_3_ cells) and synergizes erlotinib by targeting optineurin (OPTN)-dependent cell cycle arrest. Development of PAWI-2 as an anti-PC drug candidate addresses an unmet clinical need. PAWI-2 may also improve standard of care for patients because it synergizes eradication of hPCSCs.

## Results

### Effect of PAWI-2 on cell viability and self-renewal capacity of FGβ_3_ cells

FGβ_3_ cells were generated by stable transfection of fast-growing (FG) human PC cells with human β_3_/pcDNA3.1^19^. FGβ_3_ cells possessing CSC-like properties have an elevated expression of integrin β_3_ compared to parental, bulk FG cells (Fig. 1B). PAWI-2 was two-fold more potent to inhibit cell viability (Supplemental Table S1, Fig. 1C) of FGβ_3_ cells (IC_50_, 15 nM) compared to FG cells (IC_50_, 36 nM). FGβ_3_ cells showed four-fold increased self-renewal capacity (i.e., secondary tumor sphere formation mediated by integrin β_3_) relative to FG cells (Fig. 1D-F). PAWI-2 (20 nM) inhibited self-renewal capacity two-fold in FGβ_3_ cells (Fig. 1E). *In vitro*, PAWI-2 was two-fold more effective to inhibit self-renewal capacity of FGβ_3_ (IC_50_, 16 nM) compared to FG cells (IC_50_, 31 nM) (Fig. 1F, Supplemental Table S1).

### Effect of PAWI-2 on induction of mitochondrial-controlled apoptosis

PAWI-2 potently (i.e., 5.9-fold relative to vehicle-control) activated apoptosis (i.e., activation of caspase-3/7, Fig. 1G,H) in FGβ_3_ cells (EC_50_, 11 nM, 48 hours). PAWI-2-mediated apoptosis was less apparent in FG cells (EC_50_, 42 nM; 3.5-fold increase). Selective potency of PAWI-2 was further shown by PARP cleavage. Compared to FG cells, induction of PARP cleavage was more apparent in FGβ_3_ cells (6.7- vs. 3.2-fold increase, respectively, at 24 hours; Fig. 1I). Similarly, apoptosis induced by PAWI-2 in FG and FGβ_3_ cells was controlled by ATM-mitochondrial p53-dependent apoptotic signaling. PAWI-2 activated upstream DNA-damage checkpoint via ATR/ATM-kinase activation (Supplemental Fig. S1A) and inhibited cytosolic p53/Bax binding to anti-apoptotic Bcl-xL (Supplemental Fig. S1B). This caused activation of pro-apoptotic p53/Bax and induced mitochondrial cytochrome c release to trigger cell apoptosis (Supplemental Fig. S1C). This mechanism of action has been observed for PAWI-2 in other non-CSC cancer cells^22–25^.

### Effect of PAWI-2 on downstream of the KRAS-NF-κB pathway

**Figure 2:**
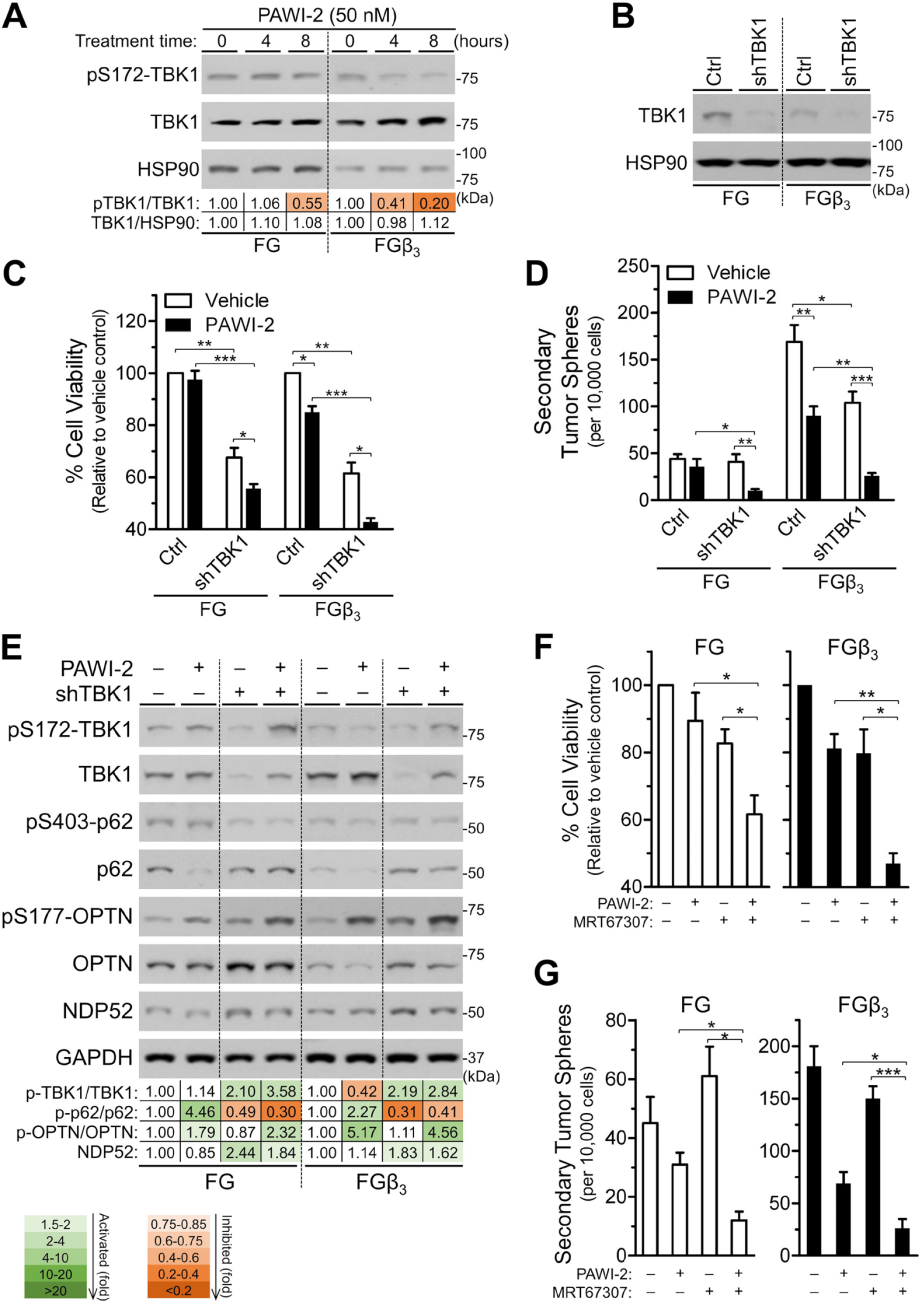
PAWI-2 affects KRAS-NF-κB signaling by targeting TBK1 phosphorylation to overcome tumor stemness. (**A**) Immunoblots and densitometry analysis of phospho-Ser172-TBK1 (pS172-TBK1) and TBK1 as determined with whole-cell extracts. (**B–-E**) TBK1 knockdown enhanced the effect of PAWI-2 in FG and FGβ_3_ cells: (**B**) immunoblots show TBK1 genetic knockdown efficiency used in this study; effect of TBK1 knockdown (**C**) on cell viability inhibited by PAWI-2 as measured by a CellTiter-Glo assay and (**D**) effects on self-renewal capacity inhibited by PAWI-2 as measured by quantifying the number of secondary tumor spheres; (**E**) immunoblots and densitometry analysis of the effect of PAWI-2 on pS172-TBK1, TBK1, phospho-Ser403-p62 (pS403-p62), p62, phospho-Ser177-OPTN (pS177-OPTN), OPTN, or NDP52 in cells with TBK1 knockdown compared to control cells. (**F**,**G**) Enhancement of inhibition of (**F**) cell viability and (**G**) self-renewal capacity by co-treatment of PAWI-2 with TBK1 specific inhibitor (MRT67307, 1 µM). Concentrations of PAWI-2 used were as indicated: 50 nM in **A**, **E**, 10 nM in **C**, **F** and 20 nM in **D**, **G**; treatment time used was as indicated: 0–16 hours in **A**, 24 hours in **C**, **D**, **F**, **G** and 8 hours in **E**; vehicle control (0.5% DMSO). GAPDH or HSP90 was used as a loading control in **A**, **B**, **E**. Data are mean ± SD (n = 3) in **C**, **D**, **F**, **G**; *P*-values were estimated by Student *t* tests in **C**, **D**, **F**, **G** (*P < 0.05, **P < 0.01, ***P < 0.001). The full-length blots are presented in Supplementary Fig. S7.

In FGβ_3_ cells, overexpression of integrin α_v_β_3_ interacts with KRAS through galectin-3 to recruit KRAS and RalB to activate TBK1 and NF-κB that triggers dysregulated KRAS-RalB-NF-κB signaling^19–21^. This is the dominant mechanism to induce CSC-like properties in FGβ_3_ cells and causes drug resistance for established FGβ_3_ tumors. Accordingly, the effect of PAWI-2 on dysregulated integrin α_v_β_3_-KRAS-NF-κB signaling in FGβ_3_ cells was studied. PAWI-2 neither caused disruption of KRAS interactions with other effectors (i.e., integrin β_3_, galectin-3; Supplemental Fig. S2A) nor inhibited Ral GTPase (i.e., did not affect RalA/B-GTP, an active form of RalA/B; Supplemental Fig. S2B). PAWI-2 inhibited KRAS-NF-κB phosphorylation of TBK1 at Ser172 (pSer172-TBK1; Fig. 2A) without affecting other key components of this pathway (integrin β_3_, KRAS, galectin-3, RalB and c-Rel were not affected by PAWI-2 up to 5 µM; Supplemental Fig. S2C). PAWI-2 selectively inhibited phosphorylation of TBK1. Compared to FG cells (IC_50_, 92 nM), PAWI-2 inhibited phosphorylation of TBK1 (pSer172-TBK1/TBK1) 5-fold more potently in FGβ_3_ cells (IC_50_, 17 nM; Supplemental Fig. S2D), similar to other *in vitro* cell viability, self-renewal capacity, and cell apoptosis characterizations (10–40 nM; Fig. 1C,F,G; Supplemental Table S1).

Downstream interruption of KRAS-NF-κB signaling (i.e., inhibition of TBK1 with TBK1 shRNA; Fig. 2B-E) largely overcame integrin β_3_-mediated stemness (i.e., less tumor sphere formation in FGβ_3_ cells with TBK1 knockdown; Fig. 2D). Treatment with PAWI-2 (10–20 nM) enhanced inhibition of TBK1 knockdown on cell viability (20% greater) and self-renewal capacity (44% greater) in FGβ_3_ cells (Fig. 2C,D). TBK1 can phosphorylate p62/sequestosome-1 (p62) at Ser403 and optineurin (OPTN) at Ser177^26^. However, after treatment with PAWI-2, both phosphorylation of p62 or OPTN were increased 2–5 fold in FG and FGβ_3_ cells (Fig. 2E). Genetic knockdown of TBK1 down-regulated phosphorylation of p62 but not autophosphorylation of TBK1 or phosphorylation of OPTN (pS172-TBK1 and pS177-OPTN, respectively, Fig. 2E).

Compared to cells treated with PAWI-2 alone, co-treatment with TBK1 kinase inhibitor (MRT67307)^27^ and PAWI-2 enhanced inhibition of cell viability (30% greater; Supplemental Table S2; Fig. 2F) and self-renewal capacity (25% greater; Fig. 2G) in FG and FGβ_3_ cells. In FGβ_3_ cells, enhancement of cell killing with co-treatment with PAWI-2 and MRT67307 was not associated with induction of cell apoptosis. For example, in the presence of PAWI-2 and MRT67307, caspase activation and PARP cleavage was comparable to treatment of PAWI-2 alone (Supplemental Fig. S3A,B). The enhanced inhibition of cell viability and self-renewal capacity (Fig. 2F,G) was associated with OPTN phosphorylation (2–4 fold activation; Supplemental Fig. S3C) similar to the result observed in the genetic knockdown of TBK1 (2–5 fold activation; Fig. 2E). Moreover, pharmaceutical inhibition of TBK1 by MRT67307 also down-regulated phosphorylation of p62 (pS403-p62) but not pS172-TBK1 or pS177-OPTN (Supplemental Fig. S3C). This result showed that phosphorylation of p62 induced by PAWI-2 was most likely related to TBK1 activity but phosphorylation of OPTN may not be solely associated with TBK1 activity.

### Effect of PAWI-2 on OPTN phosphorylation in the presence of other inhibitors

**Figure 3:**
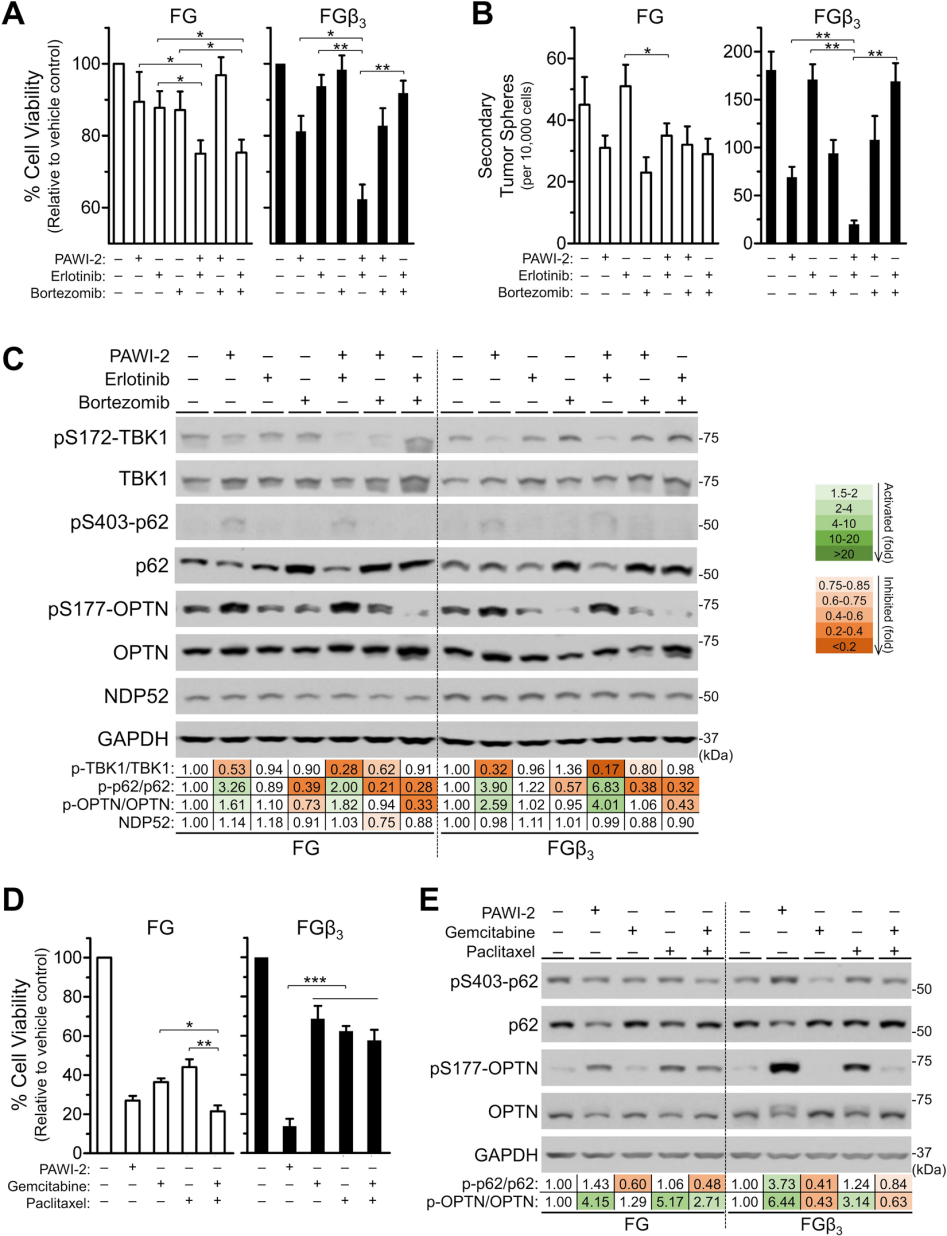
(**A–C**) PAWI-2 overcomes erlotinib resistance in FGβ_3_ cells. Inhibition of (**A**) cell viability and (**B**) self-renewal capacity (secondary tumor sphere formation) by EGFR inhibitor (erlotinib) in the presence of PAWI-2 is significantly enhanced compared to single agent treatment or combination of erlotinib (1 µM) with proteasome inhibitor (bortezomib). (**C**) Immunoblots and densitometry analysis of the effect on phospho-Ser403-p62 (pS403-p62), p62, phospho-Ser177-OPTN (pS177-OPTN), OPTN, or NDP52 in FG and FGβ_3_ cells after co-treatment of PAWI-2 with erlotinib or bortezomib. (**D**) PAWI-2 is more effective than clinically-approved drug combinations of gemcitabine (25 nM) with paclitaxel (25 nM) in FGβ_3_ cells at inhibiting cell viability; (**E**) Immunoblots and densitometry analysis of the effect of gemcitabine or paclitaxel or in combination on pS403-p62, p62, pS177-OPTN, OPTN compared to PAWI-2 alone. Concentrations of PAWI-2 used were as indicated: 10 nM in **A**, 20 nM in **B**, 50 nM in **C-E**; concentrations of bortezomib used were as indicated: 10 nM in **A**, 20 nM in **B**, 50 nM in **C**; treatment time used was as indicated: 72 hours in **A**, **D**, 24 hours in **B**, 8 hours in **C** and 16 hours in **E**; vehicle control (0.5% DMSO). GAPDH was used as a loading control in **C**, **E**. Data are mean ± SD (n = 3) in **A**, **B**, **D**; *P*-values were estimated by Student *t* tests in **A**, **B**, **D** (*P < 0.05, **P < 0.01, ***P < 0.001). The full-length blots are presented in Supplementary Fig. S7.

Integrin β_3_-mediated self-renewal capacity is associated with drug resistance in FGβ_3_ cells^19^. Co-administration of erlotinib with proteasome inhibitor bortezomib was examined to determine effects on cell viability (Supplemental Table S2) and self-renewal capacity^19,21^. In FGβ_3_ cells, co-administration of “PAWI-2 and erlotinib” enhanced inhibition of erlotinib alone on cell viability (30% greater) and self-renewal capacity (80% greater), compared to co-administration of “erlotinib and bortezomib” (Fig. 3A,B). Chou-Talalay analysis of synergism or antagonism was calculated based on a dose-dependent inhibition of cell viability for drug alone or drug-drug combinations. Synergism or antagonism between drugs was defined by combination index (CI values), showing PAWI-2 synergized erlotinib (but antagonized bortezomib, CI values > 1; Table 1) with greater synergism for FGβ_3_ cells compared to FG cells (CI values < 1; Table 1). Synergism between “erlotinib and bortezomib” was observed in FG cells but was less apparent in FGβ_3_ cells (Table 1). Synergism of “PAWI-2 and erlotinib” paralleled induction of cell apoptosis (i.e., “PAWI-2 and erlotinib” enhanced activation of caspase and PARP cleavage compared to PAWI-2 or erlotinib alone; Supplemental Fig. S4A,B).

**Table 1:** Combination index (CI) values quantified synergism after treatment with PAWI-2 and erlotinib or bortezomib in FG and FGβ_3_ cells.

Cell lines	Drug/Combo^a^	CI^b^ values at different EDs^c^		
		ED_75_	ED_90_	ED_95_
FG	Erlotinib + Bortezomib	**0.51**^d^	**0.56**^d^	**0.59**^d^
	Erlotinib + PAWI-2	**0.64**^d^	**0.74**^d^	**0.86**^d^
	Bortezomib + PAWI-2	1.56	1.50	1.47
FGβ_3_	Erlotinib + Bortezomib	**0.87**^d^	1.07	1.19
	Erlotinib + PAWI-2	**0.45**^d^	**0.32**^d^	**0.25**^d^
	Bortezomib + PAWI-2	1.55	1.59	1.63

^a^Ratios of Erlotinib:Bortezomib, Erlotinib:PAWI-2 and Bortezomib:PAWI-2 were 50:1, 50:1 and 1:1, respectively;

^b^Combination Index (CI) values were calculated based on the Chou-Talalay method; values of CI < 1, = 1 and > 1 indicate synergism, additive and antagonism, respectively;

^c^ED_75, 90, 95_ represent concentrations that cause 75%, 90% and 95% of proliferation inhibition, respectively;

^d^Bold values show synergy.

Bortezomib works on inhibition of late stage autophagy that promotes accumulation of p62^28^. However, in our hands, the effect of bortezomib on autophagy alone or in combination with erlotinib in FG and FGβ_3_ cells was modest (p62 and LC3 accumulation was < 2-fold; Fig. 3C, Supplemental Fig. S4C). The distinct pattern of changes of LC3-I to LC3-II was not significantly affected by co-treatment of PAWI-2 with erlotinib. This showed synergism of PAWI-2 with erlotinib was not dominated by an autophagy-related effect. Synergism between PAWI-2 and erlotinib and antagonism between PAWI-2 with bortezomib were highly correlated with OPTN phosphorylation based on a plot of CI values versus pS177-OPTN fold-change (correlation coefficient r^2^ > 0.8). Co-administration of erlotinib and PAWI-2 increased pS177-OPTN 4-fold in FGβ_3_ cells. In contrast, in the presence of PAWI-2 and bortezomib, OPTN phosphorylation was at control value. Similar results were observed for p62 and this can be explained because OPTN acts like a p62-like receptor^29^.

Combination chemotherapy of gemcitabine and *nab*-paclitaxel has been widely used in the treatment of advanced PC^30,31^. This drug combination showed comparable inhibition of FG cell viability with PAWI-2 alone (Supplemental Table S3; Fig. 3D). However, in FGβ_3_ cells, co-administration of gemcitabine and paclitaxel did not show significant enhancement on inhibition of cell viability compared to gemcitabine or paclitaxel alone (Fig. 3D). Drug resistance of this combination in FGβ_3_ cells was not associated with activated apoptosis because a comparable effect (activation on caspase activity and PARP cleavage for the combination compared to gemcitabine or paclitaxel alone; Supplemental Fig. S5A,B) was observed in both FG and FGβ_3_ cells. Synergism between gemcitabine and paclitaxel was associated with OPTN phosphorylation (Fig. 3E). OPTN phosphorylation may be linked to microtubule (MT) disturbance because this effect was also observed in paclitaxel (MT stabilizer)-treated cells (Fig. 3E).

### Effect of PAWI-2 and MT disturbance agents on cell cycle arrest

**Figure 4:**
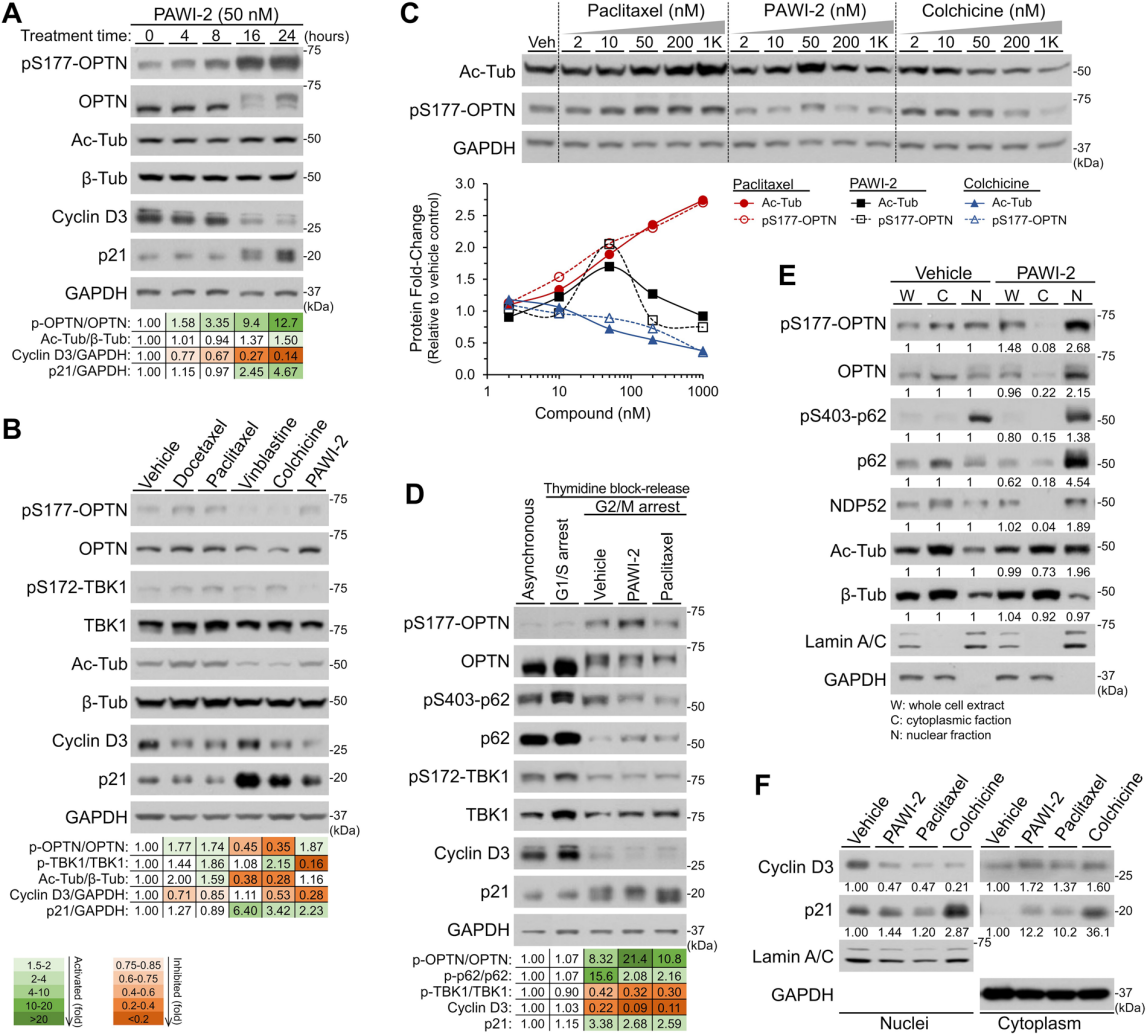
OPTN-dependent signaling controlled cell cycle arrest and the effect of PAWI-2 to overcome tumor stemness in FGβ_3_ cells. (**A**) Time-dependent effect of PAWI-2 on phosphorylation of OPTN (pS177-OPTN/OPTN) correlated with tubulin acetylation (Ac-Tub/β-Tub), cyclin D3 decrease and p21 phosphorylation. (**B**) The effect of MT disturbance agent (stabilizer: docetaxel, paclitaxel; destabilizer: vinblastine, colchicine) on pS177-OPTN, OPTN, pS172-TBK1, TBK1, Ac-Tub, β-Tub, cyclin D3 or p21 compared to PAWI-2 alone. (**C**) Dose-dependent response of paclitaxel, PAWI-2, or colchicine on pS177-OPTN and Ac-Tub. (**D**) FGβ_3_ cells arrested at the G1/S boundary with a double thymidine block and then released into fresh medium containing PAWI-2 or paclitaxel. Immunoblots and densitometry analysis of protein markers in G2/M arrest cells were done for comparison. (**E**) FGβ_3_ cells were fractionated into cytoplasmic (C) and nuclear (N) fractions. Immunoblots were conducted for indicated protein markers and compared to whole cell extracts (W). (**F**) The effect of PAWI-2, paclitaxel or colchicine on cytoplasmic accumulation of p21 and decrease of cyclin D3 in nuclear fractions. Concentrations of PAWI-2 used were as indicated: 50 nM in **A**, **B**, **D-F**, 2–1000 nM in **C**; concentrations of other MT disturbance agents used were as indicated: 50 nM in **B**, **D**, **F**, 2–1000 nM in **C**; treatment time used was as indicated: 0–24 hours in **A**, 8 hours in **B-F**; vehicle control (0.5% DMSO). GAPDH was used as a loading control of whole cell extract in **A**-**D** and a marker of cytoplasmic fraction in **E**, **F**; Lamin A/C was used as a marker of the nuclear fraction in **E**, **F**. The full-length blots are presented in Supplementary Fig. S7.

PAWI-2 binds tubulin at the same site as colchicine^22^. Changes in pS177-OPTN and acetylation of tubulin (related to MT stabilization) as a function of PAWI-2 treatment in FGβ_3_ cells was evaluated (Fig. 4A). OPTN phosphorylation was correlated with tubulin acetylation and cell cycle arrest indicators (i.e., 4–6 fold decrease of cyclin D3 and 2–4 fold increase of p21 phosphorylation; Fig. 4A). For comparison, several well-defined MT disrupting agents (including MT stabilizers docetaxel and paclitaxel; MT destabilizers vinblastine and colchicine; Fig. 4B) confirmed this effect. Activation or inhibition of pS177-OPTN closely paralleled increase or inhibition of tubulin acetylation (Fig. 4B). Dose-dependent responses on OPTN phosphorylation or tubulin acetylation (Fig. 4C) by treatment with paclitaxel (activation of pS177-OPTN), PAWI-2 (MT stabilizer or destabilizer, dose-dependent changes on pS177-OPTN) and colchicine (inhibition of pS177-OPTN) was observed. However, inhibition of phosphorylation of TBK1 was only observed for PAWI-2 (Fig. 4B).

Double thymidine block arrests cells at the G1/S boundary and subsequent release to fresh media arrests cells at different boundaries^32^. Experiments were done to synchronize FGβ_3_ cells at the G1/S boundary and release upon treatment with vehicle control, PAWI-2 or paclitaxel (Fig. 4D). Activation of pS177-OPTN was detected at later G2/M phase (8 hours after release). This was closely associated with onset of cyclin D3 degradation and also inhibition of TBK1 phosphorylation (Fig. 4D). Similar results were observed for phosphorylation of p62 on Ser403 (pS403-p62). Based on intracellular distribution studies in FGβ_3_ cells, OPTN and pS177-OPTN (and also p62, NDP52) were mainly found in the cytoplasmic fraction under vehicle control conditions but accumulated in the nucleus with PAWI-2 (Fig. 4E). Accumulation of OPTN in the nuclear fraction was an indicator of G2/M arrest^33^. Cellular trafficking mediated by PAWI-2 was also associated with acetylated tubulin localization to nuclei (Fig. 4E). Similarly, nuclear cyclin D3 downregulation and accumulation of p21 (and its phosphorylated form) in cytoplasm were observed after administration of PAWI-2, paclitaxel or colchicine to FGβ_3_ cells (Fig. 4F), providing strong evidence that these MT disturbing agents caused FGβ_3_ cell G2/M arrest^34,35^. Together, these data show that PAWI-2 induced OPTN phosphorylation was highly associated with cell cycle arrest during mitosis.

## Discussion

**Figure 5:**
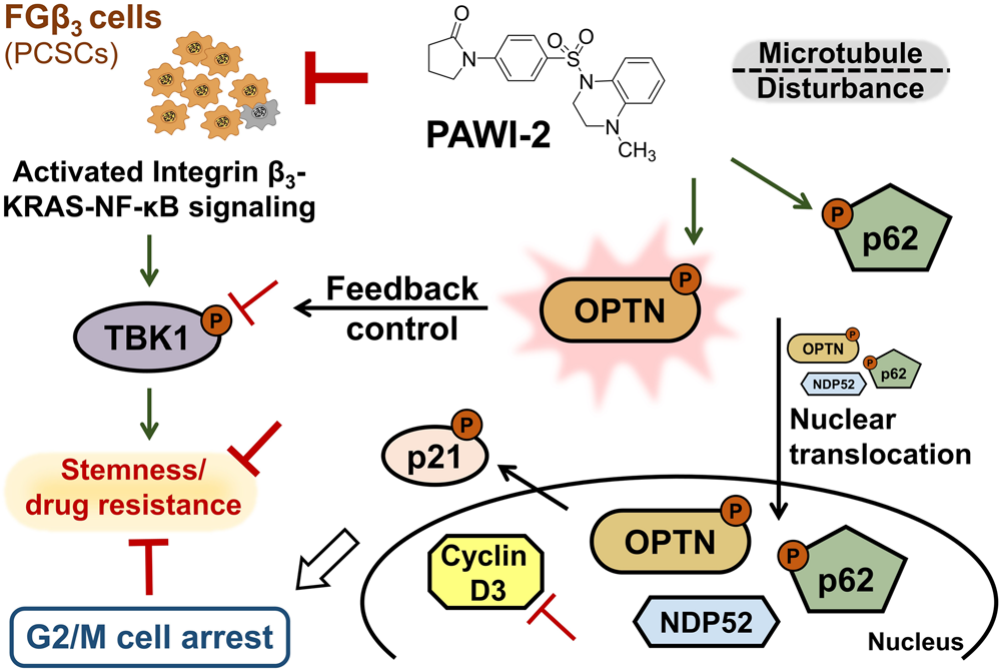
Proposed model depicts a mechanism of PAWI-2 to overcome tumor stemness and drug resistance in FGβ_3_ cells. Green arrows, stimulation; red lines, inhibition.

We have shown that PAWI-2 could reverse cancer stemness and overcome drug resistance in an integrin β_3_ KRAS-dependent hPCSCs (i.e., FGβ_3_ cells). A working model of PAWI-2 was proposed (Fig. 5). In this model, OPTN plays a central role in regulation of TBK1 functional activity to reverse tumor stemness and drug resistance in FGβ_3_ cells. Phosphorylation of conserved OPTN residue (Ser177) by PAWI-2 promotes OPTN translocation into the nucleus and causes G2/M arrest. Concomitantly, OPTN phosphorylation induced by PAWI-2 has negative feedback control on TBK1 (dephosphorylation of TBK1 at S172) to inhibit dysregulation of KRAS-NF-κB signaling in FGβ_3_ cells. This model links a role of OPTN to the functional interplay between G2/M cell cycle arrest and provides a mechanism to explain how PAWI-2 overcomes tumor stemness.

Previously, we showed PAWI-2 activated DNA-damage checkpoint and mitochondrial p53-dependent apoptotic signaling in other non-CSC cancer cells^22–24^. Data herein showed this was also observed for hPCSCs (FGβ_3_ cells). For dysregulated KRAS-RalB-NF-κB signaling in FGβ_3_ cells, galectin-3 plays a critical role in clustering integrin α_v_β_3_ to induce KRAS and enable multiple processes in cancer cells^21^. In the study herein, PAWI-2 did not disrupt KRAS interactions with other effectors. This differentiates PAWI-2 from other drugs (e.g., GCS-100), that act as galectin-3 inhibitors and pharmacologically disrupt biochemical association between integrin α_v_β_3_ and KRAS^21^. RalA/B serves as molecular regulators of integrin α_v_β_3_-KRAS-NF-κB signaling. PAWI-2 also did not measurably affect the inactive/active forms of RalA/B. These findings suggest that PAWI-2 inhibited KRAS-NF-κB signaling regardless of KRAS or Ral status. Given the fact that >90% of KRAS is activated by mutations in PC^36^ and RAS or Ral inhibitors of these pathways have not proven effective clinically^19^, this suggests that PAWI-2 may possess advantages in clinical applications.

TBK1 is a serine/threonine kinase that is activated by autophosphorylation at Ser172 within the kinase activation loop^37^. Association of TBK1 with RalB of the major oncogene (RAS) in the integrin α_v_β_3_-KRAS-NF-κB signaling pathway promotes tumorigenesis^19,21^. TBK1 inhibitors (e.g., momelotinib) show limited utility in PC even in combination with other effective PC therapeutics^38^. As a key kinase in several signaling pathways, TBK1 also phosphorylates p62 or OPTN to enhance their binding capacity with poly-ubiquitin (poly-UB) chains^26,39^. TBK1 constitutively interacts with OPTN to act as a key modulator to initiate elimination of damaged mitochondria via selective mitophagy (PINK1/Parkin-dependent mitophagy), that is involved in tumor suppression pathways^40,41^. PAWI-2 was previously reported to affect mitochondrial function (i.e., membrane trafficking, mitochondrial membrane potential changes)^23,24^. However, in FGβ_3_ cells neither PINK1 nor Parkin proteins were altered by PAWI-2. This data excludes mitophagy mechanisms initiated via OPTN by PAWI-2. PAWI-2 did not change autophagy biomarker LC3-I to lipidated form LC3-II (Supplemental Fig. S4C). Activation of OPTN phosphorylation by PAWI-2 may be related to other signaling cascades not solely dependent on TBK1. OPTN has also been shown to directly regulate TBK1^42^. A negative feedback control of TBK1 activation by OPTN helps explain the proposed working mechanism of PAWI-2. PAWI-2-induced OPTN phosphorylation negatively regulates TBK1 functional activity (i.e., autophosphorylation inhibited), and causes inhibition of KRAS-NF-κB signaling. This was further shown by exacerbated effects of PAWI-2 on the action of genetic knockdown of TBK1 and pharmacological inhibition (MRT67307) of TBK1 activation. Interestingly, MRT67307 does not affect accumulation of pS172-TBK1 (reversely activated). This shows that in contrast to previous reports^27,43^, TBK1 activation may not be the sole autocatalytic mechanism responsible operating for MRT67307.

In addition to being a downstream regulator of TBK1 function, OPTN is involved in a variety of other biological functions, including protection against apoptosis, Golgi organization, exocytosis, antiviral innate immune response, selective autophagy and other membrane trafficking mechanisms^29,41^. OPTN does not have any reported enzymatic activity but usually acts as an adaptor protein that links two different proteins (e.g., TBK1 and PINK1/Parkin)^29,41^. For tumorigenesis or tumor stemness, OPTN phosphorylation has been largely attributed to regulation of mitophagy^44,45^ mediated by TBK1, but that was not observed herein for PAWI-2. Phosphorylation of OPTN at Ser177 also plays a pivotal role in mitotic progression and induces OPTN translocation into the nucleus^46^. OPTN-dependent G2/M cell cycle arrest induced by PAWI-2 in FGβ_3_ cells parallels this process. Previously, G2/M arrest was independently observed in PAWI-2-treated colon cancer cells^22^. This regulatory mechanism is abolished at the end of the G2/M phase as a consequence of nuclear translocation of OPTN and leads to increased activity of TBK1 (Supplemental Fig. S6**)**.

Synergism between PAWI-2 and other validated drugs (i.e., erlotinib) was controlled by phosphorylation of OPTN. In contrast, in FGβ_3_ cells, if antagonism was observed (e.g., PAWI-2 with bortezomib), phosphorylation of OPTN was abolished. This observation helps explain drug resistance observed for FGβ_3_ cells treated with well-documented PC chemotherapies (e.g., gemcitabine with paclitaxel, Fig. 3E)^30,31^. OPTN may work as an over-arching branch-point for PAWI-2 inhibition of cell viability to overcome self-renewal capacity in FGβ_3_ cells and also to synergize other pathway inhibitors (i.e., erlotinib).

In PC cells, PAWI-2 binds to tubulin to stabilize/destabilize microtubules (MTs) and activate apoptotic signaling^22–24^. Phosphorylation of OPTN was closely associated with MT stabilization because this effect was also observed in cells treated with other MT stabilizers (e.g., paclitaxel or docetaxel; Fig. 4B). OPTN foci distribution is dependent on the integrity of MTs^46,47^, but no relationship between OPTN phosphorylation and MT disturbance has been reported thus far. Nothing describing synergism between clinically-validated cancer drugs through regulation of OPTN has been reported. Accumulation of pS177-OPTN in the presence of MT stabilizers may be due to the essential role of MTs in coordinating and organizing many crucial cellular steps^48^. Thus, OPTN phosphorylation induced by PAWI-2 or other MT stabilizers could modulate synergism effects to overcome drug resistance and combat more aggressive CSCs.

In conclusion, PAWI-2 synergized specific pathway inhibitors (e.g., TBK1 inhibitors, EGFR inhibitors) against CSCs. Selective pharmacological potency of PAWI-2 in CSCs (e.g., FGβ_3_ cells versus FG cells) showed the utility of PAWI-2 to inhibit CSCs versus bulk cancer cells. This observation provides a basis for PAWI-2 as an efficient treatment of PC, especially in highly aggressive/metastatic cancer with stem-like properties and intrinsic or acquired drug resistance.

## Methods

### Cell lines

FG and FGβ_3_ cells were provided by Dr. David Cheresh (UC San Diego and The Scripps Research Institute). FGβ_3_ cells have been thoroughly documented as an aggressive cell line showing CSC-like properties and cancer drug resistance^19–21^. FG and FGβ_3_ cells were grown in DMEM with 10% FBS. After thawing, cell lines were cultured at 37 °C in a humidified 5% CO_2_ atmosphere and routinely screened for mycoplasma contamination.

### Compounds

Synthesis and pharmaceutical properties of PAWI-2 (Fig. 1A) were reported previously^25,49^. Other drugs/inhibitors used in this study are listed in the Supplementary Materials and Methods.

### Cell viability and apoptosis assays

FG and FGβ_3_ cells were seeded onto plates and treated with test compounds (vehicle, 0.5% DMSO; PAWI-2 or other drugs; 2 to 5000 nM) for 3 days. Cell viability was determined using CellTiter-Glo (Promega). Data were expressed as percentage of survival compared to survival of vehicle-treated cells. A similar protocol was used to test synergy of PAWI-2 in the presence of erlotinib and/or bortezomib. Chou-Talalay analysis used commercial software (ComboSyn)^50^. Cell apoptosis was determined by quantifying caspase-3/7 activity using Caspase-Glo 3/7 (Promega).

### Tumor-sphere culture and self-renewal assay

FG and FGβ_3_ cells were seeded on ultra-low attachment plates at single-cell suspensions (1,000 cells ml^−1^) in DMEM/F12 medium containing insulin-transferrin-selenium (Corning) supplemented with EGF and bFGF (Gibco). Primary tumor spheres were formed after 7 days. Cells were then treated with test compounds for 24 hours. Primary tumor spheres larger than 50 µm in diameter were counted for each condition in triplicate. Single-cell suspensions were dissociated from primary tumor spheres by filtration through a 40 µm cell strainer and seeded using the same conditions. Secondary tumor spheres were formed after 7 days and treated and counted similarly as that for primary tumor spheres.

### Subcellular fractionation, immunoprecipitation and immunoblotting

Subcellular fractionation and immunoblot experiments were carried out as before^24^. Whole-cell extracts were obtained after lysis with RIPA buffer (Supplementary Materials and Methods) and subcellular fractions were obtained after homogenization in isolation buffer and centrifugation. Immunoprecipitation experiments were carried out as before with specific antibodies^24^. Protein extracts were resolved by SDS-PAGE followed by immunoblotting using antibodies specific for target proteins (Supplementary Materials and Methods). Densities of immunoblot bands were quantified using ImageJ (NIH).

### Genetic knockdown

FG and FGβ_3_ cells were transfected with TBK1 small hairpin RNA (shRNA; Dharmacon; Supplementary Table S4) using lipofectamine 3000 reagent (Invitrogen). Gene knockdown was confirmed by immunoblotting.

### Ral activation assay

Affinity pulldown assays for RalA/B were carried out following manufacturer’s instructions (Cell Biolabs). Cells were cultured in suspension and treated with vehicle or PAWI-2 (50 nM) for 8 hours. Lysate obtained was incubated with RalBP1 PBD agarose bead slurry at 4 °C for 1 hour with gentle agitation. Activated forms of Ral (GTP bound) bound to beads were collected, washed and resolved by SDS-PAGE followed by immunoblotting using RalA/B antibodies.

### Double thymidine block and release

FGβ_3_ cells were first incubated with 2 mM thymidine (Sigma) for 18 hours and released into fresh medium for 8 hours. Thymidine treatment was repeated, and a second release was conducted for 0–8 hours by releasing cells for treatment with vehicle, PAWI-2 or paclitaxel. For G1/S boundary, cells were collected at 0 hour. For the G2/M boundary studies, cells were collected at 8 hours for analysis of protein by immunoblots.

### Statistical analysis

IC_50_ and EC_50_ values were calculated using a nonlinear regression analysis (GraphPad Prism) of the mean and standard deviation (SD) or standard error of the mean (SEM) of at least triplicate samples for each biological assay. Student *t* tests were used to calculate statistical significance and a *P*-value < 0.05 was considered significant.

## Supplementary information

Supplementary information.
