# Supplementary material for: PAWI-2 overcomes tumor stemness and drug resistance via cell cycle arrest in integrin β3-KRAS-dependent pancreatic cancer stem cells
Source: Sci Rep. 2020 Jun 8;10:9162. doi: 10.1038/s41598-020-65804-5 (PMC7280251; doi:10.1038/s41598-020-65804-5)

**Supplementary Data and Methods**

**PAWI-2 overcomes tumor stemness and drug resistance via**

**cell cycle arrest in integrin β_3_-KRAS-dependent** **pancreatic cancer stem cells**

Jiongjia Cheng and John R. Cashman

*Human BioMolecular Research Institute and ChemRegen, Inc., San Diego, CA, 92121, USA*

**Table of Contents**

[Supplementary Materials and Methods 2](#_Toc38286622)

[Chemicals and Reagents 2](#_Toc38286623)

[Antibodies 2](#_Toc38286624)

[Table S1. 3](#_Toc38286625)

[Table S2. 3](#_Toc38286626)

[Table S3. 3](#_Toc38286627)

[Table S4. 3](#_Toc38286628)

[Figure S1. 4](#_Toc38286629)

[Figure S2. 5](#_Toc38286630)

[Figure S3. 6](#_Toc38286631)

[Figure S4. 7](#_Toc38286632)

[Figure S5. 8](#_Toc38286633)

[Figure S6. 9](#_Toc38286634)

[Figure S7. Original full-length Western Blot images 10](#_Toc38286635)

# Supplementary Materials and Methods

## Chemicals and Reagents

The following materials (purchased from) were used: phosphate buffered solution (PBS), Dulbecco’s phosphate buffered solution (DPBS), trypsin-EDTA, DMEM (Corning Inc., Corning, NY); Fetal bovine serum (Atlanta Biologicals, Flowery Branch, GA); protease inhibitors (Roche, Indianapolis, IN); Pierce BCA protein assay kit and ECL reagent (Thermo Scientific, Rockford, lL); Bolt 4-12% Bis-Tris Plus Gels, 10× blot MOPS running buffer (Life Technologies, Carlsbad, CA); erlontinib and MRT67307 (Cayman chemical, Ann Arbor, MI); colchicine (Chem-Impex, Wood Dale, IL); paclitaxel (LC Laboratories, Woburn, MA); gemcitabine (Combi-Blocks, San Diego, CA); bortezomib, docetaxel and vinblastine (NCI DTP Plated Compounds, 10 mM in DMSO); Triton-X-100, bovine serum albumin fraction V (BSA), EDTA, sodium chloride, nonylphenoxypolyethoxylethanol (NP-40), 3α,12α-dihydroxy-5β-cholanic acid sodium salt (sodium deoxycholate), polyethylene glycol sorbitan monolaurate (Tween-20), dodecyl sulfate sodium salt (SDS), bicine and bis-tris (Sigma-Aldrich, St. Louis, MO); and other chemicals, reagents, solvents and devices (VWR, San Diego, CA). RIPA buffer: 25 mM Tris-HCl, pH 7.6, 150 mM NaCl, 1 % NP-40, 1 % sodium doxycholate, 0.1 % SDS in the presence of 1× protease inhibitors. Isolation buffer: 225 mM mannitol, 75 mM sucrose, 50 mM HEPEs pH 7.5 in the presence of 1× protease inhibitors.

## Antibodies

The following antibodies (purchased from, #catalog number) were used: anti-p53 (Bio-Rad, #MCA1701), anti-phospho-Ser1981-ATM (Cell Signaling #5883), anti-phospho-Ser428-ATR (Cell Signaling #2853), anti-Bcl-xL (Cell Signaling #2764; Santa Cruz #sc-8392), anti-Bax (Cell Signaling #5883), anti-Cytochrome c (Santa Cruz #sc-13156), anti-COX IV (Cell Signaling #4850), anti-PARP (Cell Signaling #9532), anti-Cleaved PARP (Asp214) (Cell Signaling #5525), anti-integrin β_3_ (Santa Cruz #sc-46655), anti-KRAS (Santa Cruz #sc-30; Proteintech #12063-1-AP), anti-Galectin-3 (Santa Cruz #sc-32790), anti-RalB (Santa Cruz #sc-390108; Cell Signaling #3523), anti-phospho-Ser172-TBK1 (Cell Signaling #5483), TBK1 (Cell Signaling #3504), c-Rel (Cell Signaling #4727), anti-phospho-Ser403-SQSTM1/p62 (Cell Signaling #39786), anti-SQSTM1/p62 (Cell Signaling #8025, #88588), anti-phospho-Ser177-Optineurin (Cell Signaling #57548), anti-Optineurin (Cell Signaling #58981), anti-NDP52 (Cell Signaling #60732), anti-Cyclin D3 (Cell Signaling #2936), anti-p21/Waf1/Cip1 (Cell Signaling #2947), anti-LC3A/B (Cell Signaling #12741), anti-β-tubulin (Sigma #T8328), anti-acetylated-tubulin (Sigma #T7451), anti-HSP90α/β (Santa Cruz #sc-13119), anti-β-Actin (Cell Signaling #3700), anti-GAPDH (Santa Cruz #sc-47724).

Table S1. Effect of PAWI-2 on FG or FGβ_3_ cell viability, apoptosis, and tumor sphere formation (self-renewal capacity).

| **Cell Lines** | **Viability** | **Apoptosis** | **Tumor Sphere formation** | |
| --- | --- | --- | --- | --- |
|  |  |  | **Primary Secondary** | |
|  | IC_50_ ± SD,  nM^a^ | EC_50_ ± SD,  nM^a^ | IC_50_ ± SD,  nM^a^ | IC_50_ ± SD,  nM^a^ |
| **FG** | 36 ± 2 | 42 ± 6 | 26 ± 6 | 31 ± 4 |
| **FGβ_3_** | 15 ± 2 | 11 ± 3 | 16 ± 3 | 16 ± 2 |

^a^IC_50_ or EC_50_ is the mean ± SD of three independent determinations.

Table S2. Effect of erlotinib, bortezomib or MRT67372 on FG and FGβ_3_ cell viability.

| **Cell Line** | **Erlotinib**  IC_50_ ± SD, µM^a^ | **Bortezomib**  IC_50_ ± SD, nM^a^ | **MRT67307**  IC_50_ ± SD, µM^a^ |
| --- | --- | --- | --- |
| **FG** | 9.4 ± 3.9 | 15 ± 2 | 5.9 ± 1.6 |
| **FGβ_3_** | 32 ± 11 | 28 ± 2 | 6.8 ± 1.1 |

^a^IC_50_ was the mean ± SD of three independent determinations.

Table S3. Effect of gemcitabine, paclitaxel or a combination on FG and FGβ_3_ cell viability.

| **Cell Line** | **Gemcitabine**  IC_50_ ± SD, nM^a^ | **Paclitaxel**  IC_50_ ± SD, nM^a^ | **Gemcitabine + Paclitaxel (1:1)**  IC_50_ ± SD, nM^a^ |
| --- | --- | --- | --- |
| **FG** | 7.1 ± 1.1 | 9.9 ± 1.1 | 5.4 ± 1.3 |
| **FGβ_3_** | 30 ± 7 | 15 ± 4 | 30 ± 4 |

^a^IC_50_ was the mean ± SD of three independent determinations.

Table S4. List of shRNA used in this study

| **Target** | **shRNA** (Dharmacon) |
| --- | --- |
|  |  |
| **TBK1** | TRC TBK1 shRNA set (TRCN0000003182-6) |
| **Ctrl** | RHS4348 |
|  | RHS4080 |

Figure S1. Effect of PAWI-2 on activation of DNA damage checkpoint and mitochondrial-p53 dependent apoptosis in FG and FGβ_3_ cells. **A**) Immunoblot analysis of the effect of PAWI-2 on phospho-Ser1981-ATM (pS1981-ATM), phospho-Ser428-ATR (pS428-ATR), total p53, Bcl-xL and Bax as determined with whole-cell extracts. **B**) Effect of PAWI-2 on the interaction of p53 or Bax with Bcl-xL by immunoprecipitation with cytosolic extracts. **C**) Effect of PAWI-2 on cellular trafficking and translocation of pro-apoptotic (p53, Bax, cytochrome c) and anti-apoptotic markers (Bcl-xL) as a function of time as determined from mitochondrial and cytosolic extracts. Concentration of PAWI-2 used was 50 nM; treatment time was as indicated: 8 hours in **A**, **B**, 0-16 hours in **C**; vehicle control (0.5% DMSO). HSP90 was used as a loading control in **A**; GAPDH was used as a loading control in **C** and COX IV was used as a marker of mitochondrial fraction in **C**; n.d., not detected. The full-length blots are presented in Fig. S7.


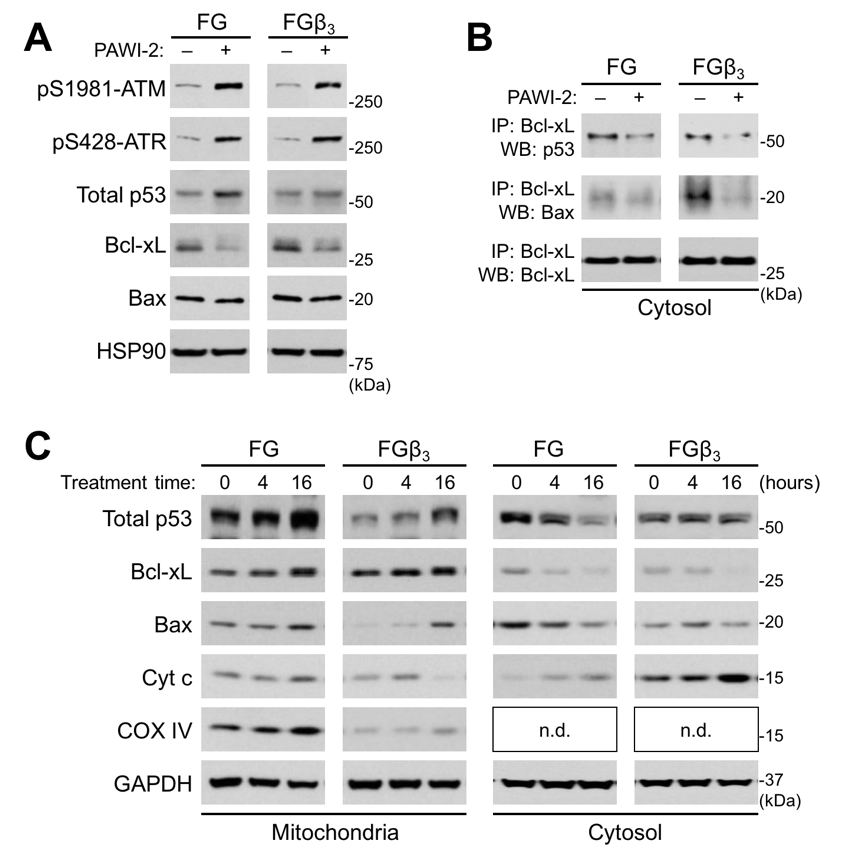


Figure S2. Effect of PAWI-2 on integrin β_3_-KRAS-NF-κB signaling in FG and FGβ_3_ cells. **A**) Effect of PAWI-2 on interaction of galectin-3 or integrin β_3_ with KRAS by immunoprecipitation of whole cell extracts. **B**) Effect of PAWI-2 on activation of RalA and RalB by immunoprecipitation of whole cell extracts with RalBP1 PBD agarose beads. **C**) Immunoblot analysis of dose-dependent effects of PAWI-2 on KRAS-NF-κB pathway markers (Integrin β_3_, Galectin-3, KRAS, RalB, phospho-Ser172-TBK1 (pS172-TBK1), TBK1 or c-Rel) as determined with whole-cell extracts. **D**) Dose-dependent inhibition of TBK1 phosphorylation by PAWI-2 in FG and FGβ_3_ cells. Concentration of PAWI-2 used was 50 nM; treatment time used was 8 hours; Veh, vehicle control (0.5% DMSO). GAPDH and HSP90 were used as loading controls. The full-length blots are presented in Fig. S7.


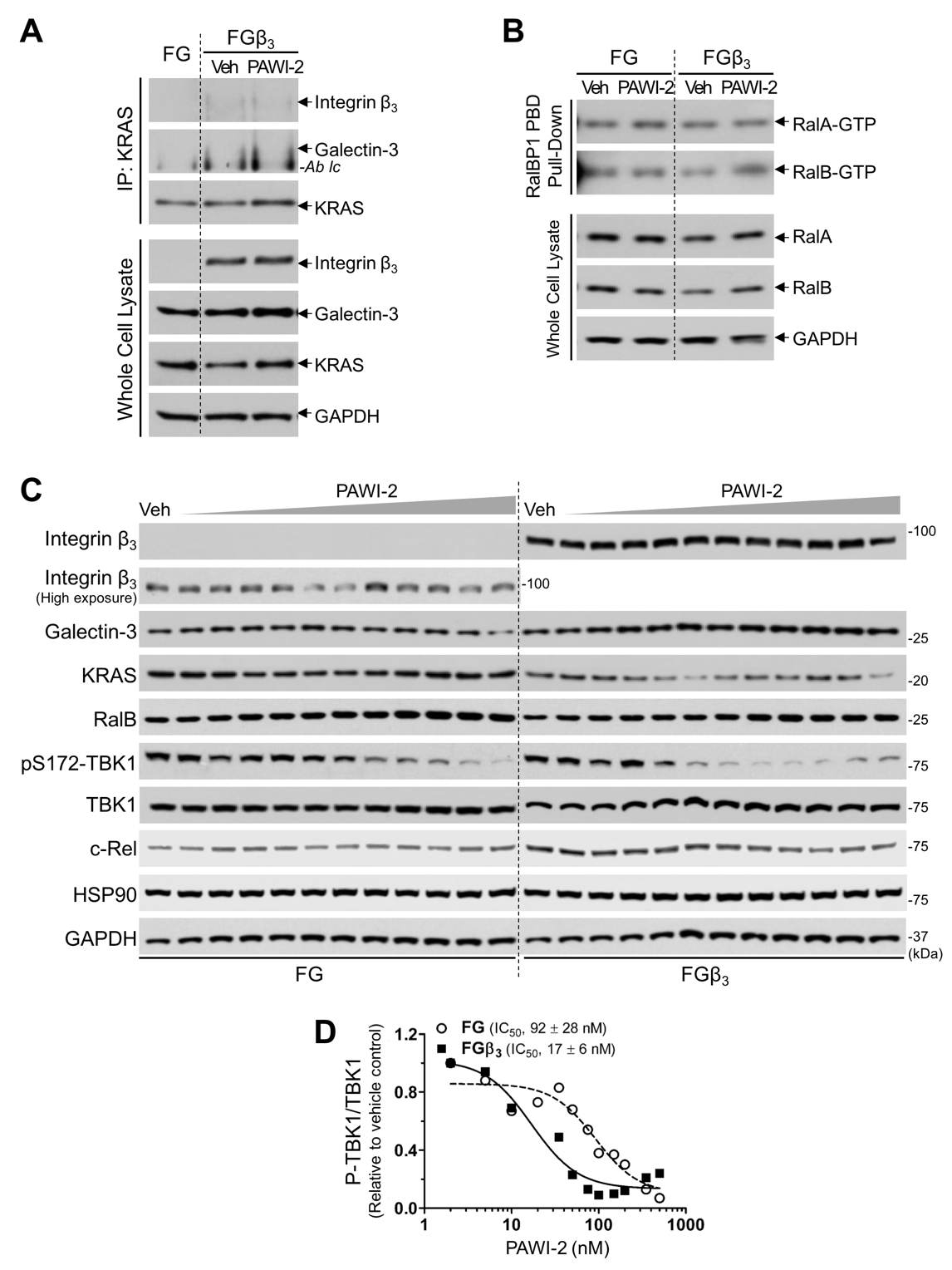


Figure S3. Effect of PAWI-2 on co-treatment of TBK1 inhibitor MRT67307 in FG and FGβ_3_ cells. **A**) Time-dependent activation of caspase-3/7 by co-treatment of PAWI-2 and MRT67307 compared to single agent treatment as determined by a Caspase-Glo 3/7 assay. **B-C**) Immunoblots and densitometry analysis of the effect of PAWI-2 on **B**) PARP (full length) or cleaved PARP, **C**) phospho-Ser172-TBK1 (pS172-TBK1), TBK1, phospho-Ser403-p62 (pS403-p62), p62, phospho-Ser177-OPTN (pS177-OPTN), OPTN or NDP52 as determined with whole-cell extracts by co-treatment of MRT67307. Concentration of PAWI-2 used was 50 nM; concentration of MRT67307 used was 1 µM; treatment time used was as indicated: 0-48 hours in **A**, 32 hours in **B** and 8 hours in **C**; vehicle control (0.5% DMSO). GAPDH or HSP90 was used as a loading control in **B**, **C**. Data are mean ± SD (n = 3) in **A**. The full-length blots are presented in Fig. S7.


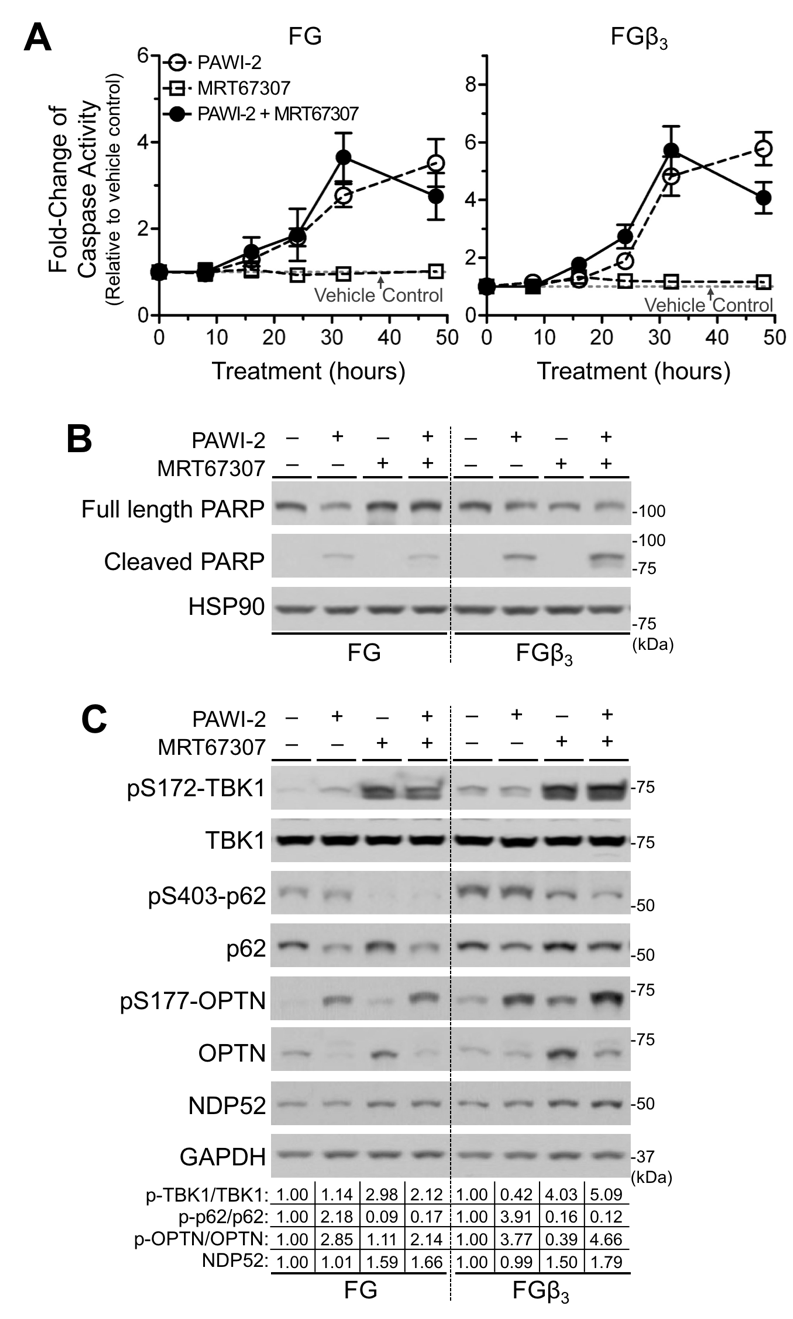


Figure S4. PAWI-2 overcomes erlotinib resistance in FGβ_3_ cells. **A**) Time-dependent activation of caspase-3/7 activity by co-treatment with PAWI-2 and erlotinib or bortezomib compared to single agent treatment as determined by a Caspase-Glo 3/7 assay. **B-C**) Immunoblots and densitometry analysis of the effect of PAWI-2 on **B**) PARP (full length) or cleaved PARP, **C**) LC-3A/B-I/-II as determined with whole-cell extracts by co-treatment of erlotinib or bortezomib. Concentration of PAWI-2 or bortezomib used were as indicated: 10 nM in **A**, 50 nM in **B**, **C**; concentration of erlotinib used was 1 µM; treatment time used was as indicated: 32 hours in **A**, 24 hours in **B**, 8 hours in **C**; vehicle control (0.5% DMSO). HSP90 or GAPDH was used as a loading control in **B**, **C**. Data are mean ± SD (n = 3) in **A**; *P*-values were estimated by Student *t* tests in **A** (**P*<0.05, ***P*<0.01). The full-length blots are presented in Fig. S7.


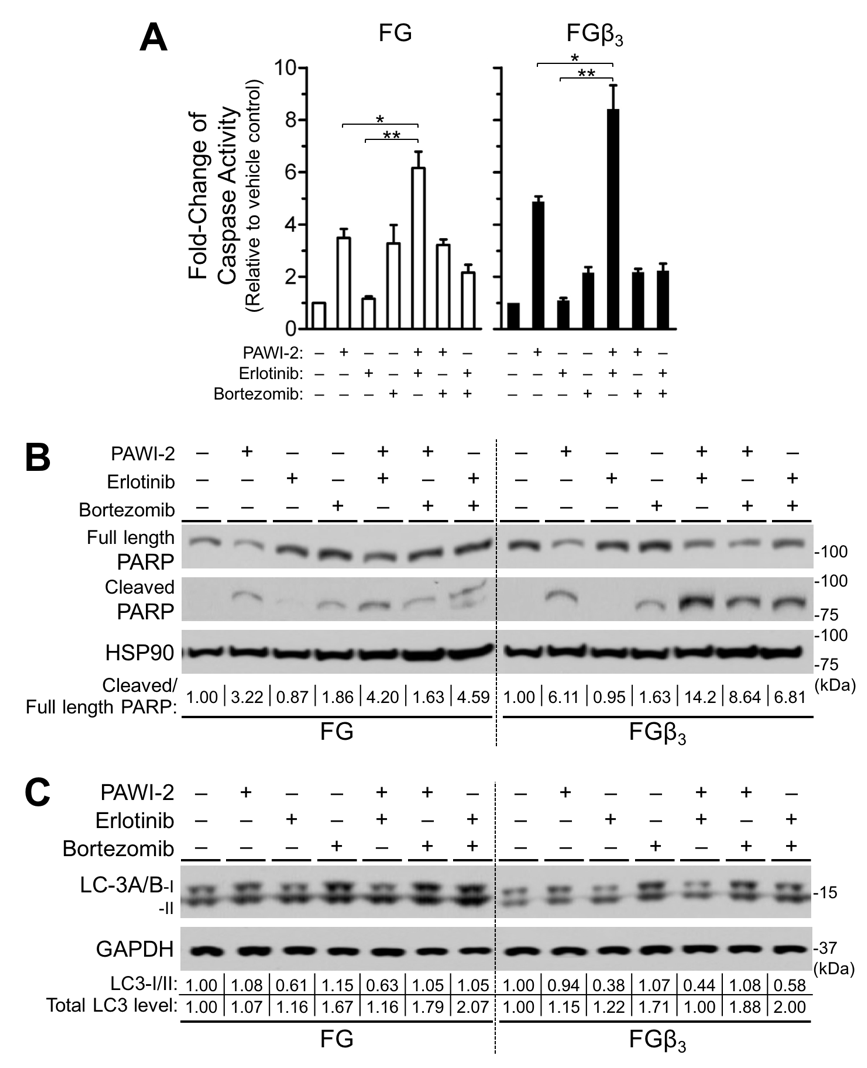


Figure S5. Activation of cell apoptosis by gemcitabine, paclitaxel or a combination in FG and FGβ_3_ cells compared to PAWI-2 alone. **A**) Time-dependent activation of caspase-3/7 activity by gemcitabine, paclitaxel or a combination compared to PAWI-2 alone as determined by a Caspase-Glo 3/7 assay. **B**) Immunoblots and densitometry analysis of the effect of gemcitabine, paclitaxel or a combination on PARP (full length) or cleaved PARP as determined with whole-cell extracts compared to PAWI-2 alone. Concentration of PAWI-2 used was 50 nM; concentration of gemcitabine or paclitaxel used was 25 nM; treatment time was as indicated: 0-48 hours in **A**, 24 hours in **B**; vehicle control (0.5% DMSO). HSP90 was used as a loading control in **B**. Data are mean ± SD (n = 3) in **A**; *P*-values were estimated by Student *t* tests in **A** (**P*<0.05, ***P*<0.01). The full-length blots are presented in Fig. S7.


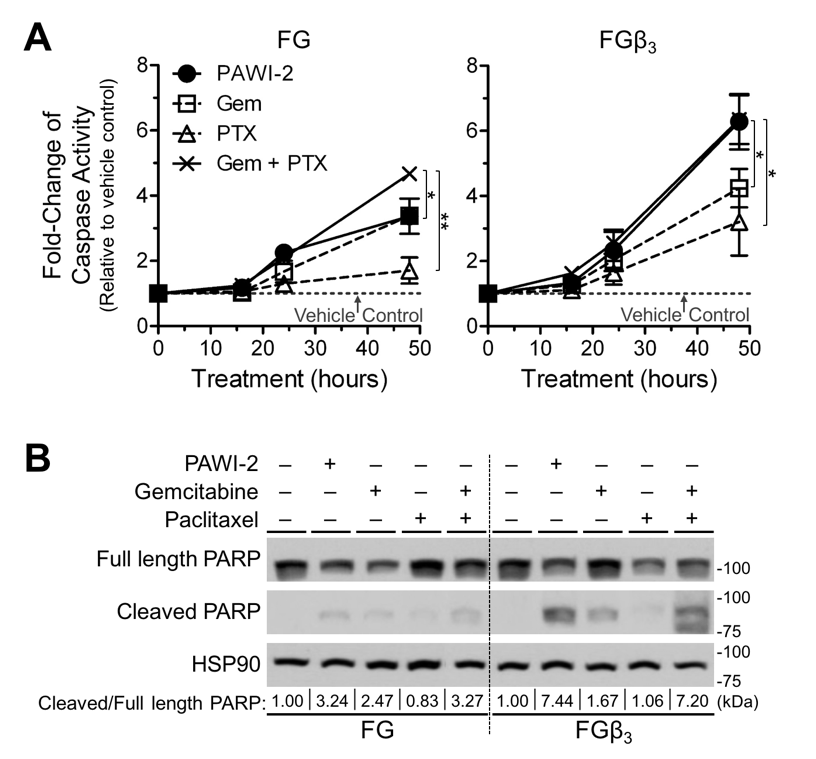


Figure S6. Time-dependent effect of PAWI-2 on phospho-Ser172-TBK1 (pS172-TBK1) or TBK1 as determined with immunoblot analysis of whole-cell extracts in FG or FGβ_3_ cells. Concentration of PAWI-2 used was 50 nM; treatment time used was 0-32 hours; vehicle control (0.5% DMSO). β-Actin was used as a loading control. The full-length blots are presented in Fig. S7.


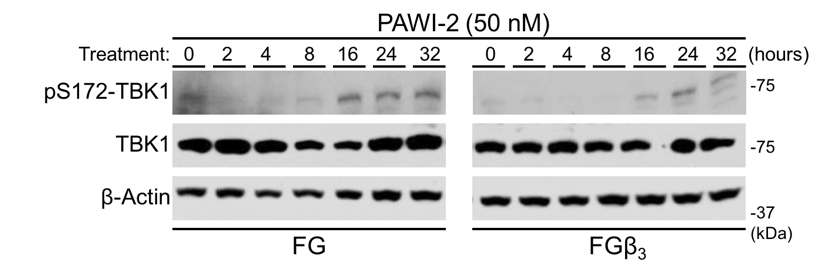


# Figure S7. Original full-length Western Blot images


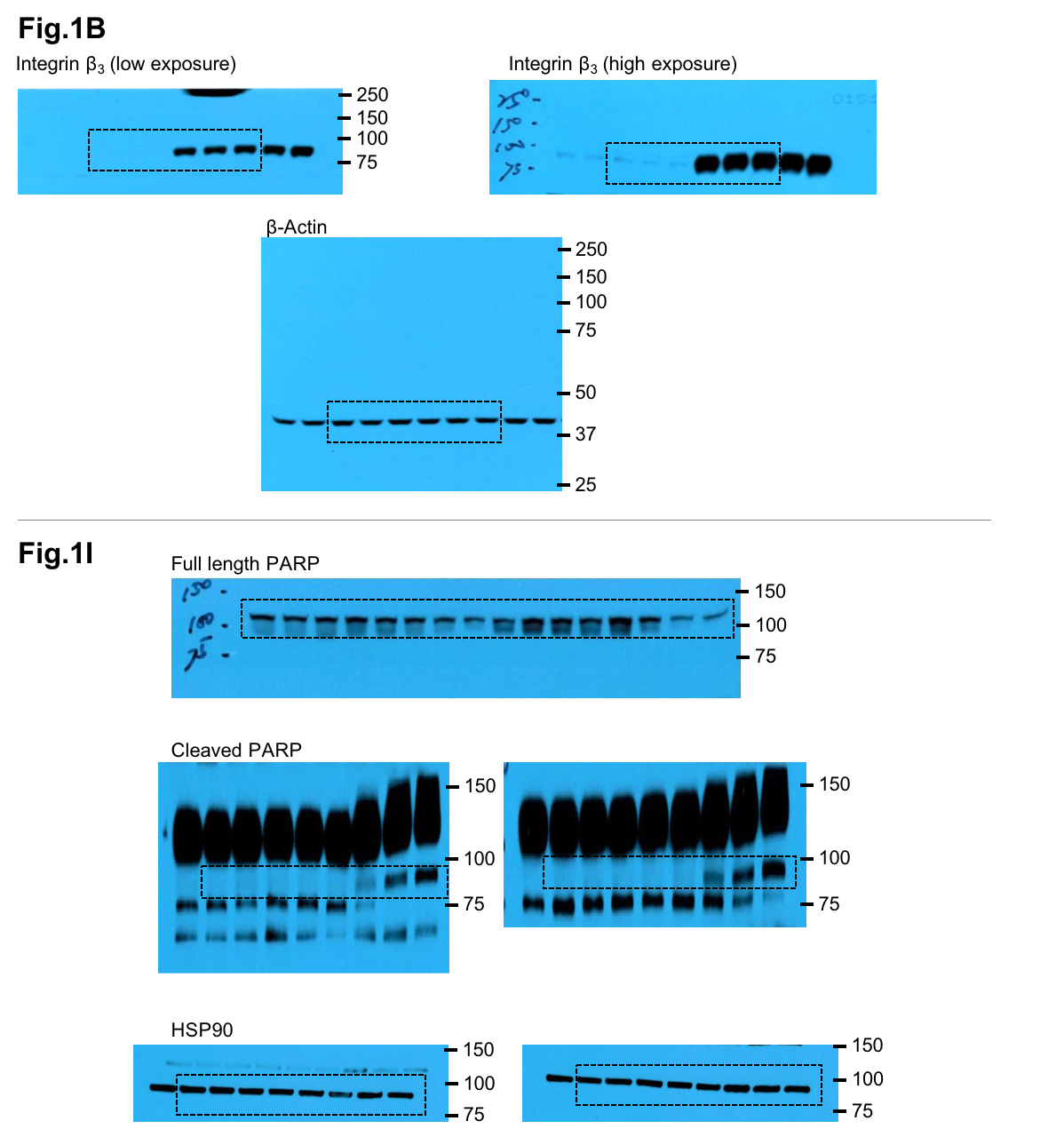


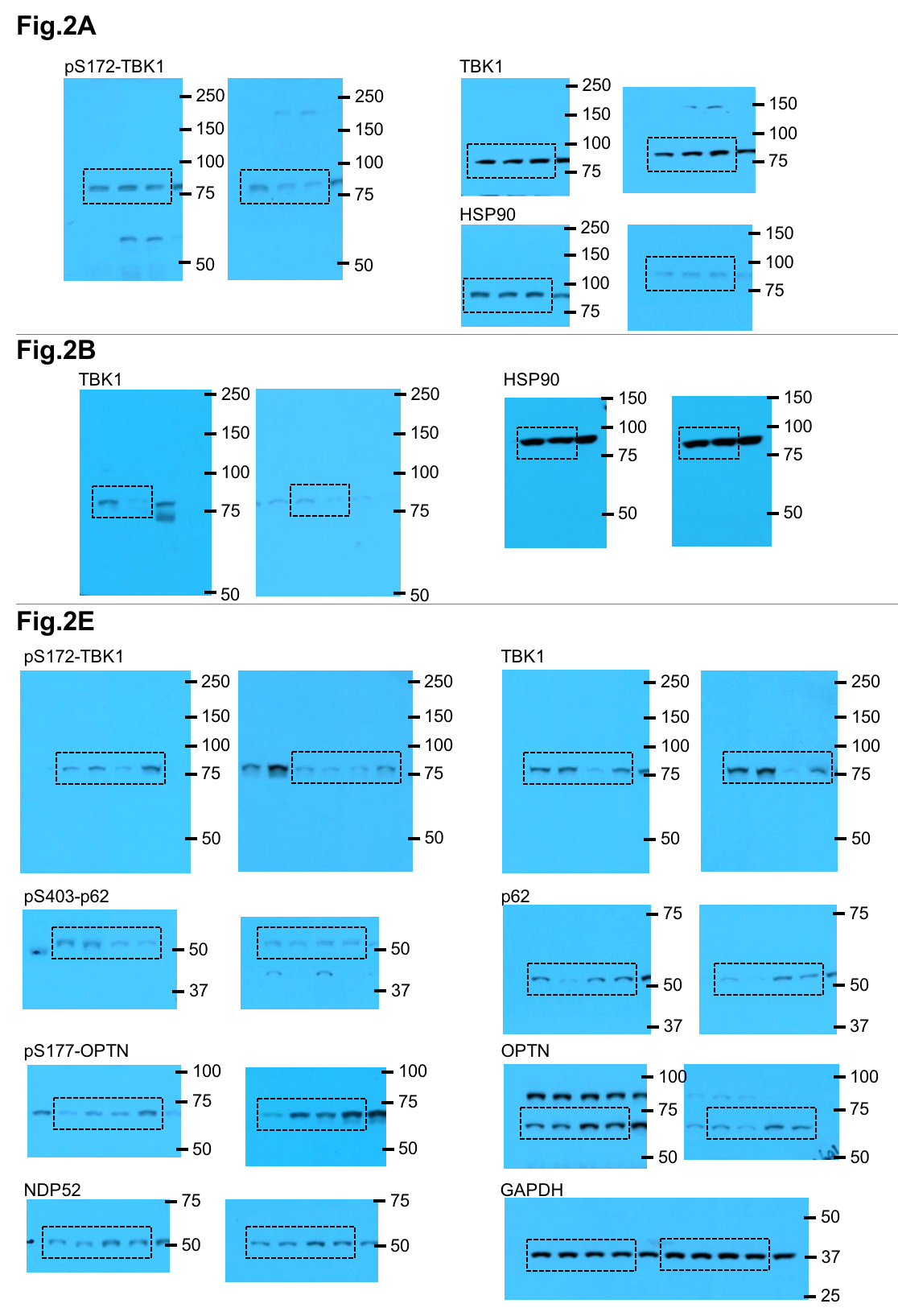


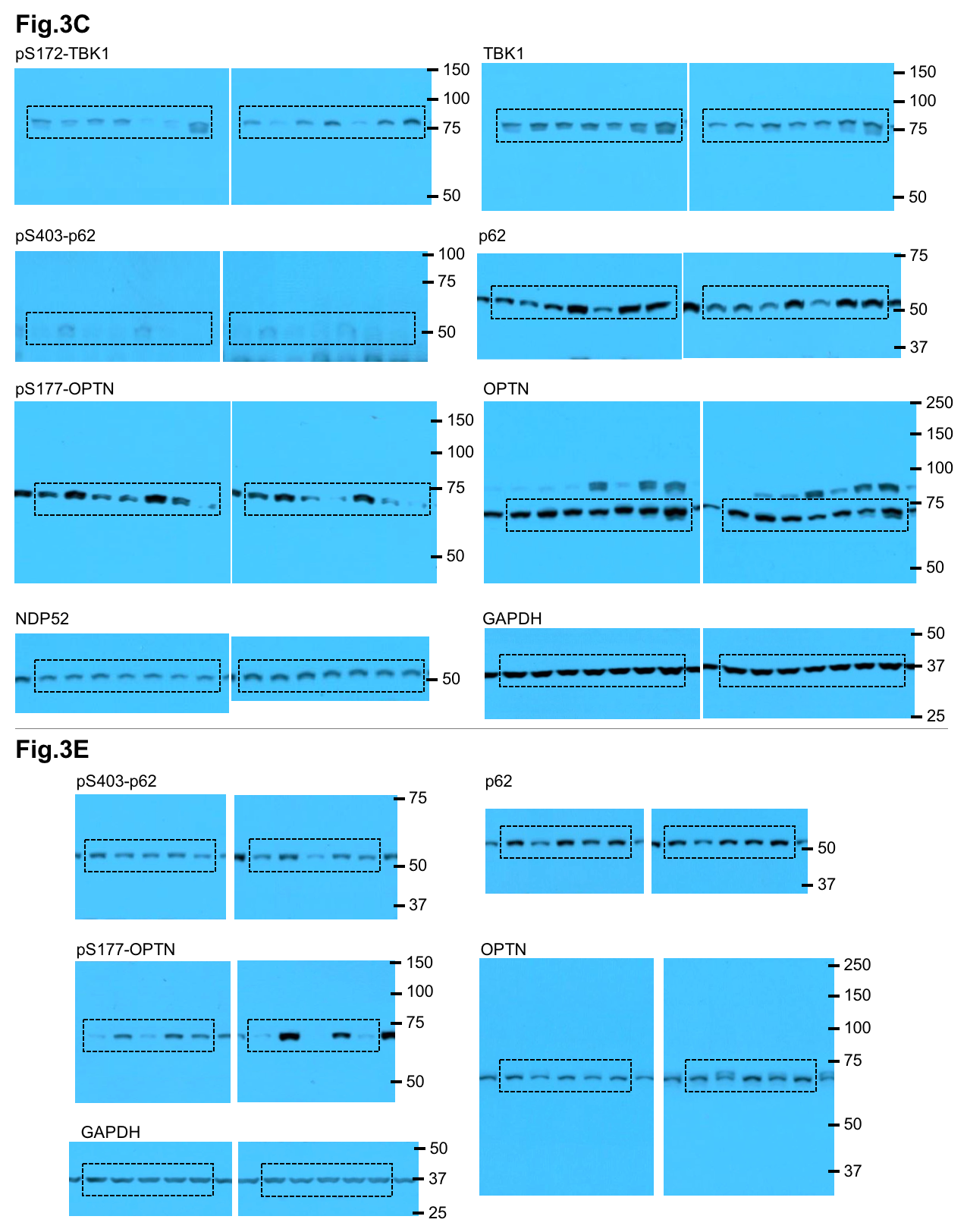


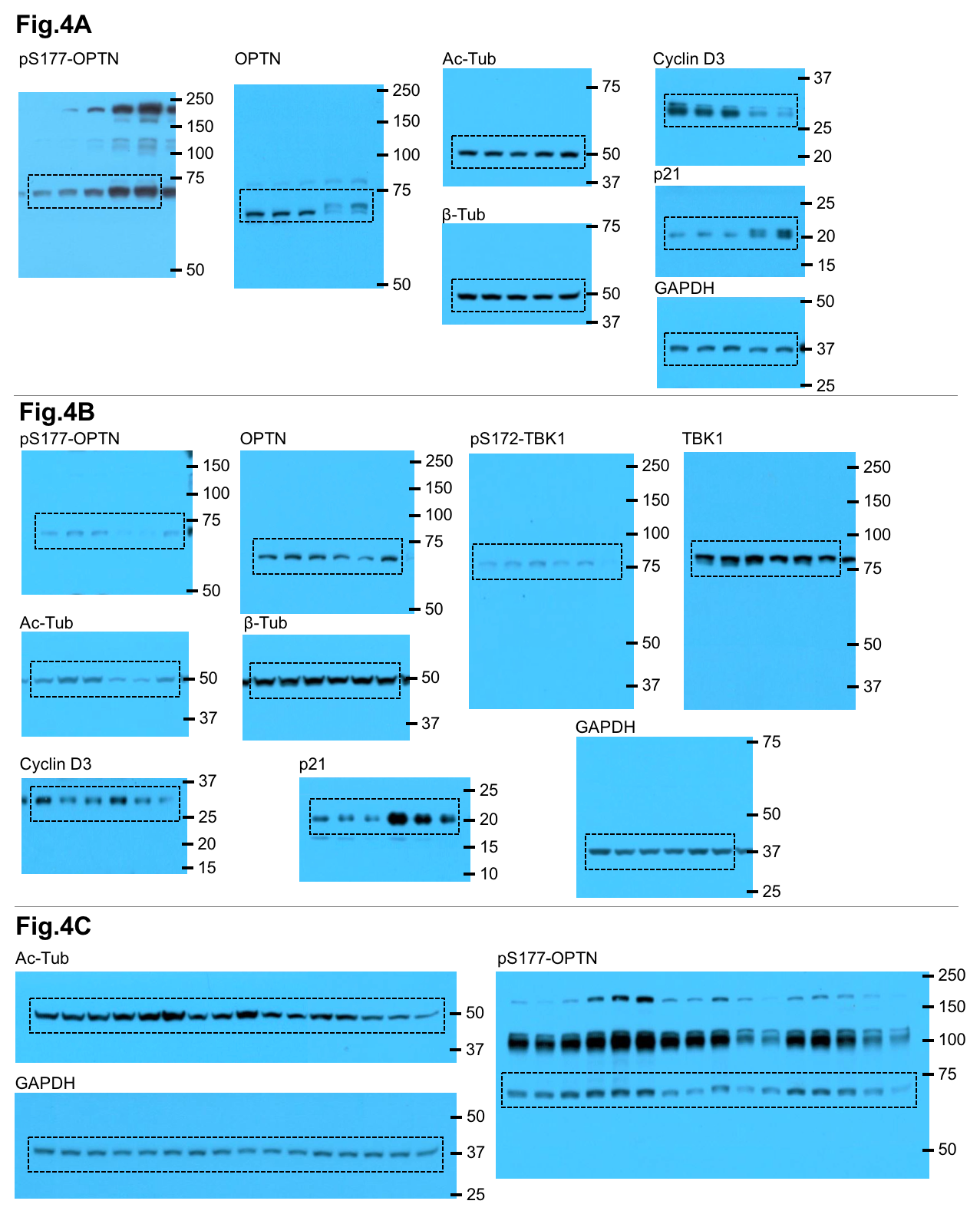


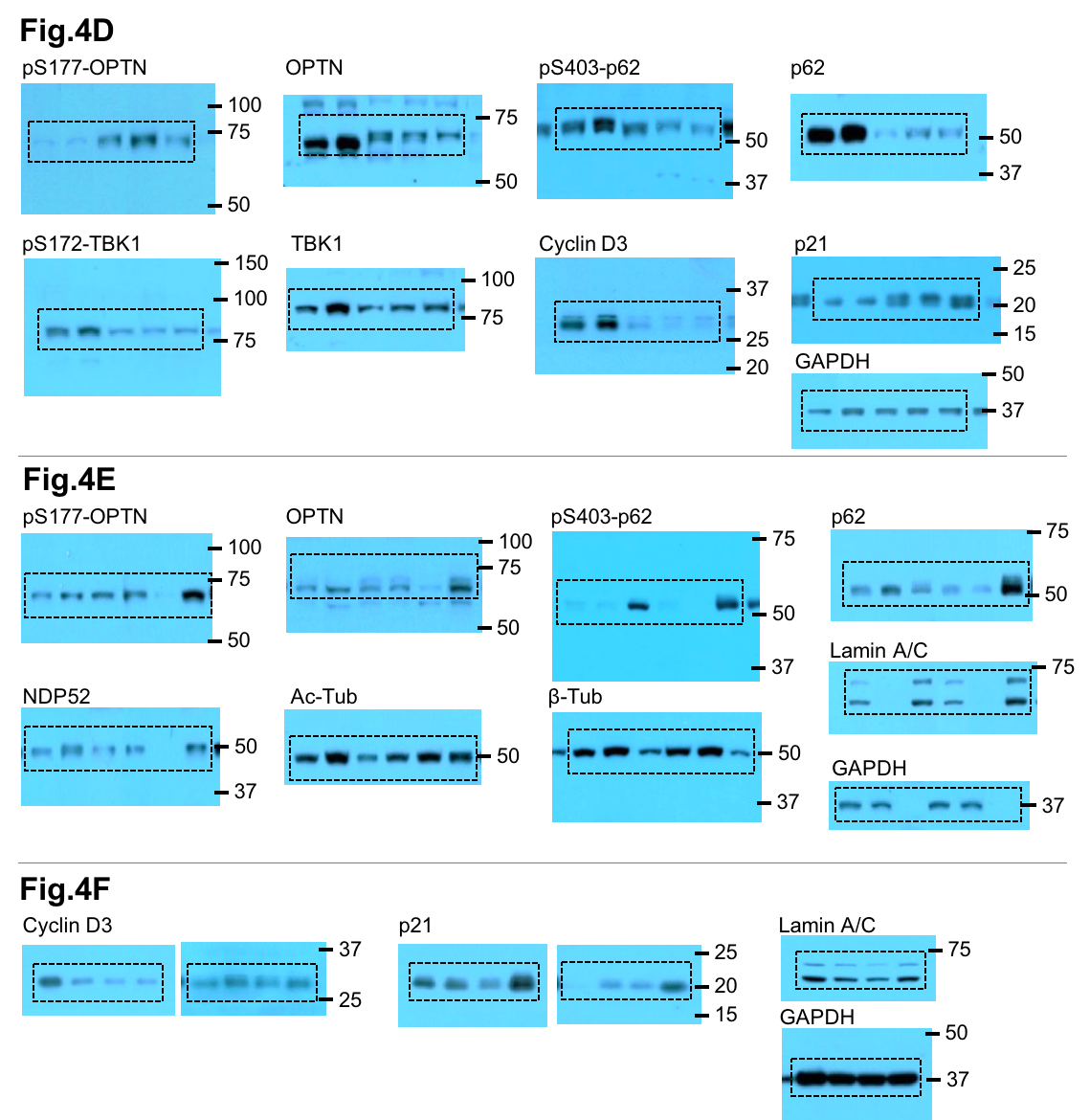


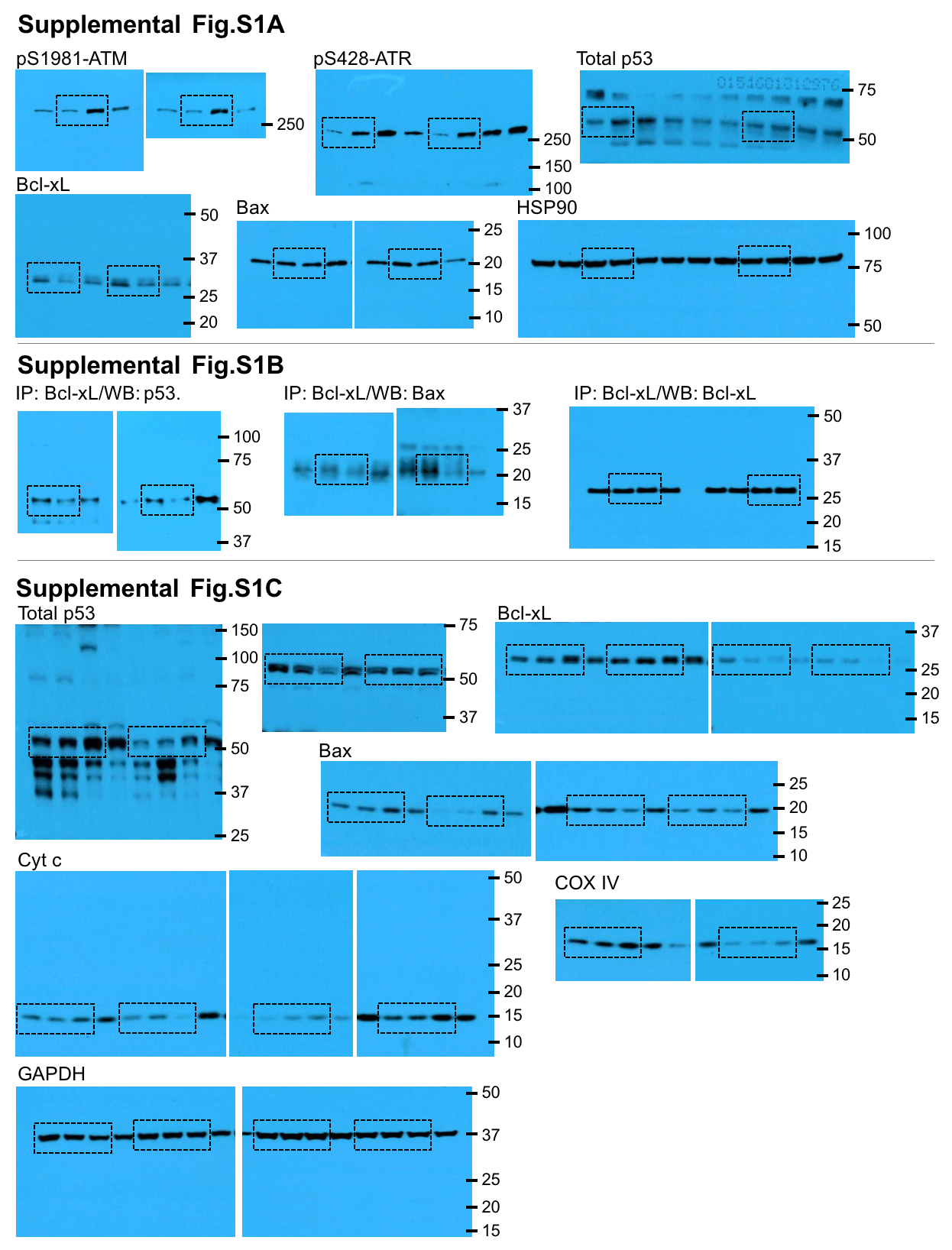


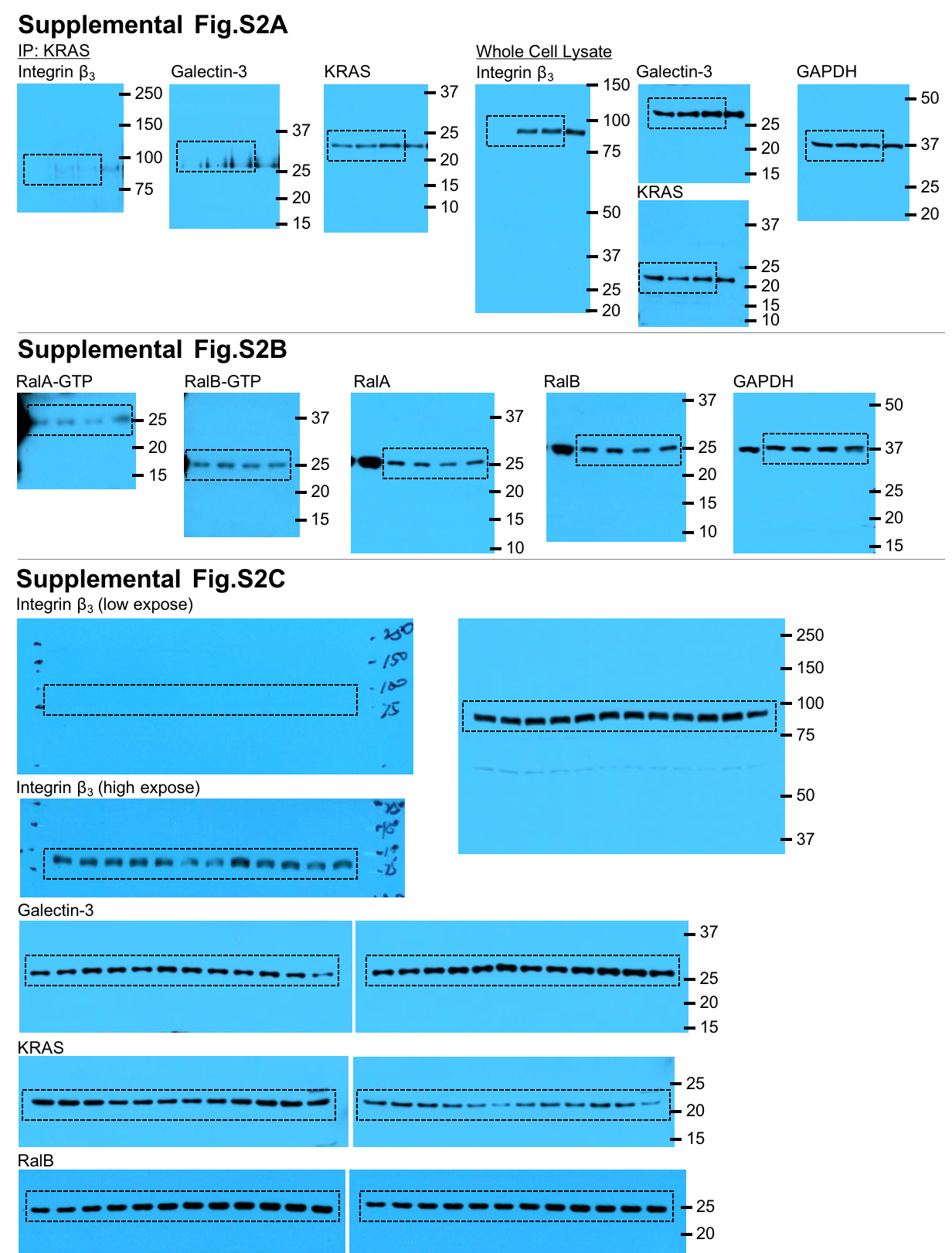


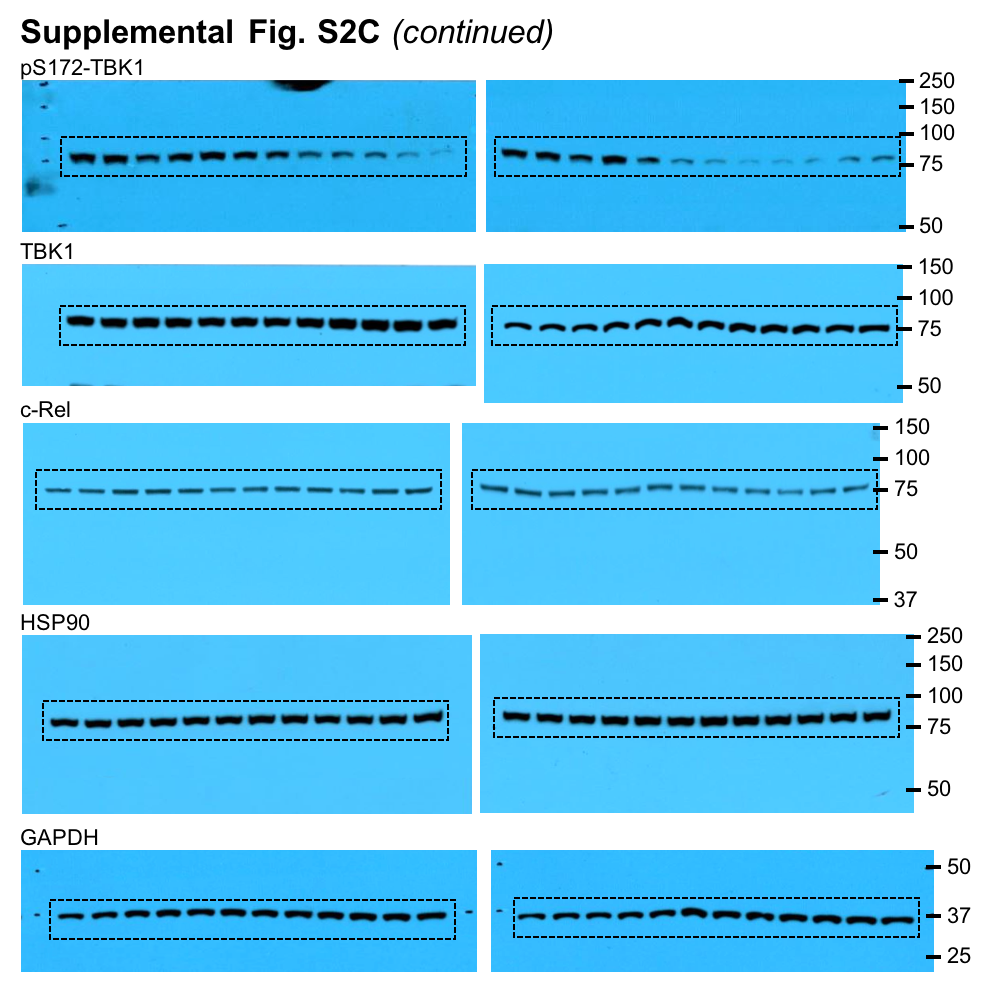


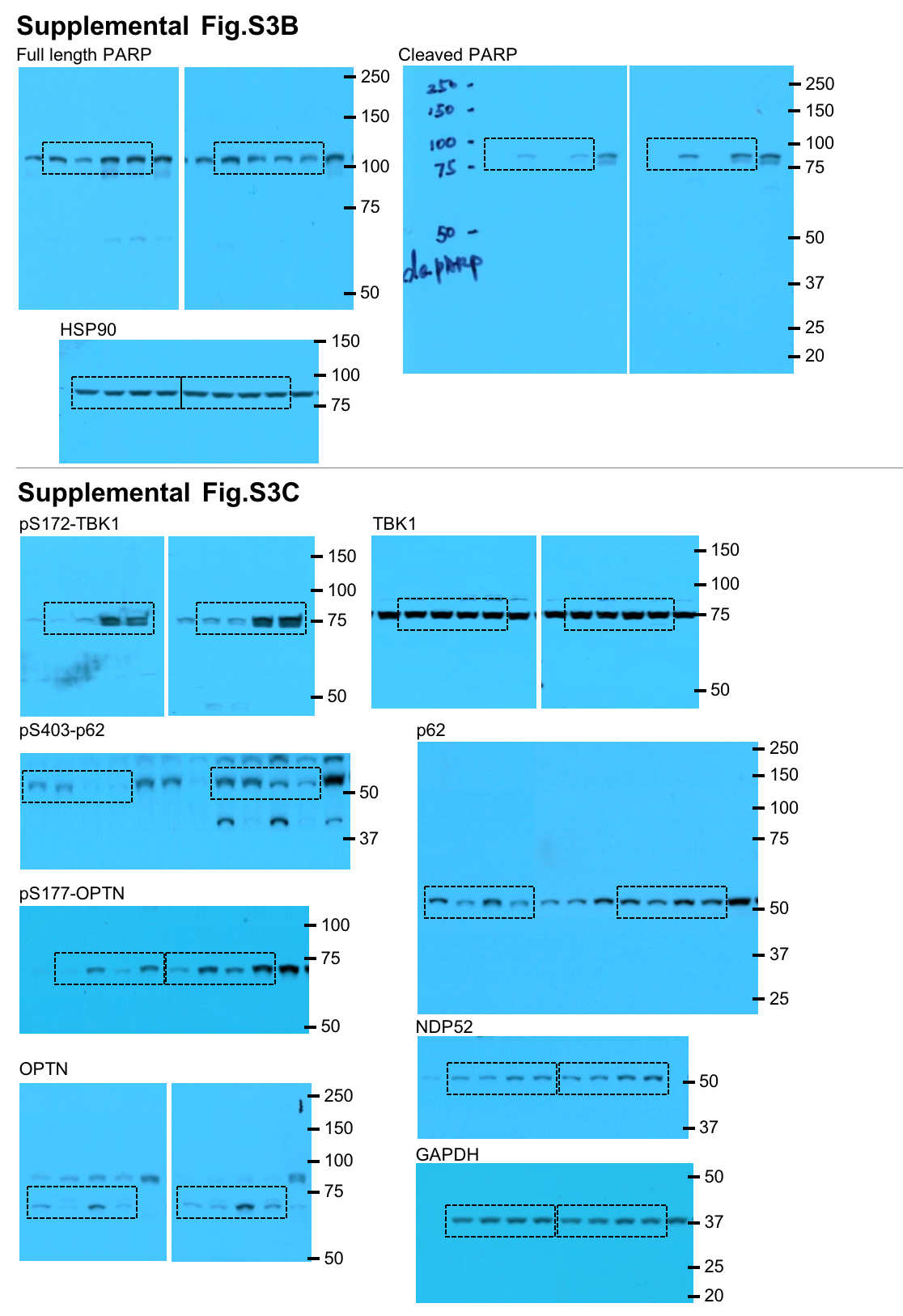


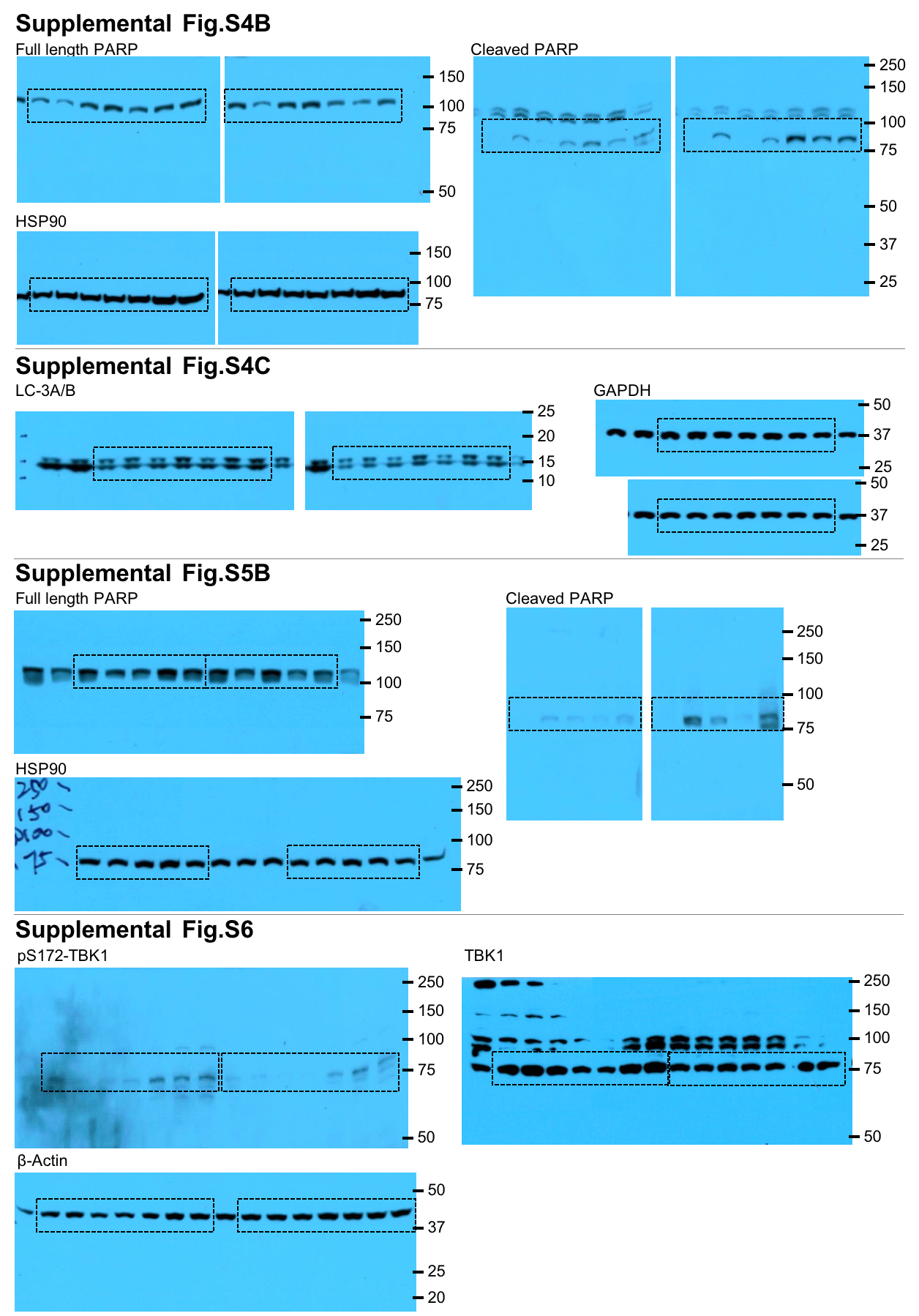

Supplement: Supplementary file 1 — Supplementary information. [file 41598_2020_65804_MOESM1_ESM.docx]
